# Targeted Hyperbranched Nanoparticles for Delivery of Doxorubicin in Breast Cancer Brain Metastasis

**DOI:** 10.1021/acs.molpharmaceut.3c00558

**Published:** 2023-11-16

**Authors:** Malcolm Lim, Nicholas L. Fletcher, Jodi M. Saunus, Amy E. McCart Reed, Haarika Chittoory, Peter T. Simpson, Kristofer J. Thurecht, Sunil R. Lakhani

**Affiliations:** † UQ Centre for Clinical Research, Faculty of Medicine, The University of Queensland, Brisbane, Herston, Queensland 4006, Australia; ‡ Centre for Advanced Imaging, The University of Queensland, Brisbane, St. Lucia, Queensland 4072, Australia; § Australian Research Council Training Centre for Innovation in Biomedical Imaging Technology, The University of Queensland, Brisbane, St. Lucia, Queensland 4072, Australia; ∥ Australian Research Council Centre of Excellence in Convergent Bio-Nano Science and Technology, The University of Queensland, Brisbane, St. Lucia, Queensland 4072, Australia; ⊥ Australian Institute for Bioengineering and Nanotechnology, The University of Queensland, Brisbane, St. Lucia, Queensland 4072, Australia; # Pathology Queensland, Royal Brisbane and Women’s Hospital, Herston, Queensland 4006, Australia

**Keywords:** breast cancer, brain metastasis, nanomedicine, hyperbranched polymer, drug delivery

## Abstract

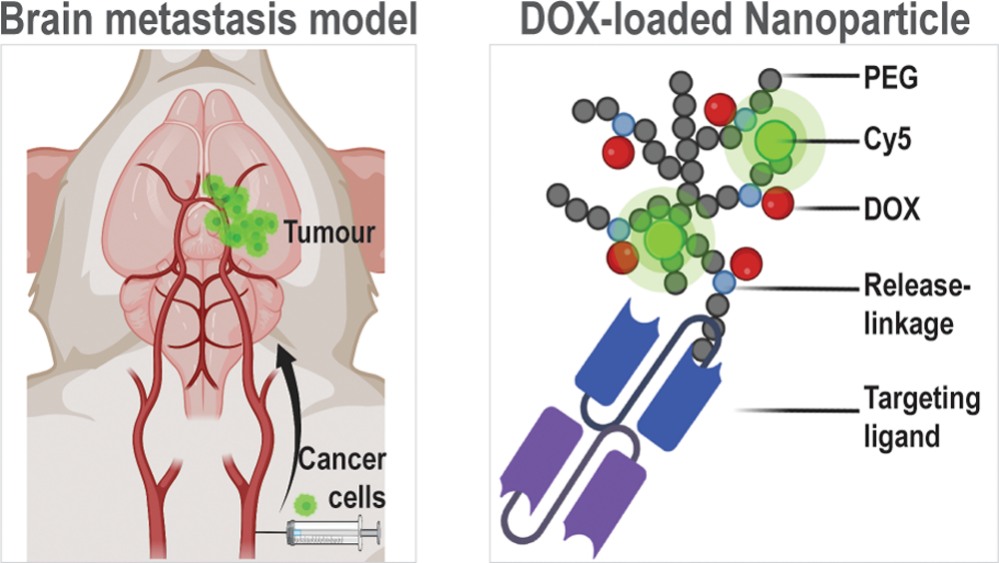

Breast cancer brain metastases (BM) are associated with a dismal prognosis and very limited treatment options. Standard chemotherapy is challenging in BM patients because the high dosage required for an effective outcome causes unacceptable systemic toxicities, a consequence of poor brain penetration, and a short physiological half-life. Nanomedicines have the potential to circumvent off-target toxicities and factors limiting the efficacy of conventional chemotherapy. The HER3 receptor is commonly expressed in breast cancer BM. Here, we investigate the use of hyperbranched polymers (HBP) functionalized with a HER3 bispecific-antibody fragment for cancer cell-specific targeting and pH-responsive release of doxorubicin (DOX) to selectively deliver and treat BM. We demonstrated that DOX-release from the HBP carrier was controlled, gradual, and greater in endosomal acidic conditions (pH 5.5) relative to physiologic pH (pH 7.4). We showed that the HER3-targeted HBP with DOX payload was HER3-specific and induced cytotoxicity in BT474 breast cancer cells (IC_50_: 17.6 μg/mL). Therapeutic testing in a BM mouse model showed that HER3-targeted HBP with DOX payload impacted tumor proliferation, reduced tumor size, and prolonged overall survival. HER3-targeted HBP level detected in ex vivo brain samples was 14-fold more than untargeted-HBP. The HBP treatments were well tolerated, with less cardiac and oocyte toxicity compared to free DOX. Taken together, our HER3-targeted HBP nanomedicine has the potential to deliver chemotherapy to BM while reducing chemotherapy-associated toxicities.

## 1 Introduction

Brain metastasis (BM) is associated with severe morbidity and high mortality. There is a high incidence of BM among breast cancer patients with primary tumors overexpressing the HER2 receptor (HER2+ breast cancers) and in triple-negative breast cancers (those lacking expression of estrogen and progesterone receptors, as well as HER2).^1^ The presence of BM is frequently diagnosed late, after presentation of neurological symptoms once the disease burden is significant in the brain.^2^

The clinical management of BM is dependent on several factors, such as the number and location of intracranial lesions, extent of extracranial disease, treatment history, and comorbidities. Therapy is therefore multimodal, consisting of neurosurgery, radiotherapy, systemic therapy, or a combination of these. Whole-brain radiotherapy is the standard of care for managing disseminated intracranial tumors.^3^ While it has been shown to improve the overall survival in BM patients,^4^ subjecting the whole brain to radiation is associated with debilitating radiotoxicity and cognitive decline.^3^ Chemotherapy is also considered on the basis of extracranial disease and treatment history and is unlikely to be indicated for the management of BM alone. However, reports have shown that intracranial tumors are as sensitive and responsive to chemotherapy as their primary tumor, suggesting combinatorial approaches are potentially efficacious and worthy of further study.^5^ Furthermore, it is known that developing intracranial BM causes disruption to vasculature and permits access and accumulation of systemic agents into the brain.^6^ Most chemotherapeutic agents are nonspecific and are associated with systemic toxicities, making it a biomedical challenge to administer a safe and efficacious concentration without harming the patient.^7^ As such, there is an urgent need for safe and effective targeted treatment options for BM with reduced off-target effects.

The concept of theranostic nanomedicine, which is based on using nanoparticles as both a probe to provide diagnostic information and a delivery system for precise drug delivery to disease sites, has the potential for managing BM. The application of nanomedicine in systemic chemotherapy involves administering the therapeutic as a nanoparticle prodrug conjugate which will selectively release its payload under specified conditions. This approach can improve the bioavailability, therapeutic window, and biodistribution of chemotherapeutic drugs while reducing nonspecific toxicities.^8^ Importantly, by reducing adverse events, there is an opportunity to improve the quality of life, as well as prolonging treatment duration for BM patients. An ideal cancer theranostic nanomedicine should include both molecular imaging probes for diagnostic feedback, as well as the ability to maintain stable systemic concentrations at a nontoxic level for an extended period of time without the need for repeated dosing.^8^ One approach is the use of an acid-hydrolyzable linkage for controlled-release of therapeutic molecules in acidic microenvironments such as within the acidic endolysosomal pathway following nanomaterial internalization.^9^

The major advantage of nanomedicine is the enhanced delivery of chemotherapy to the tumor site coupled with reduced systemic off-target exposure compared to free drug achieved through the use of nanoparticle formulation. Passive delivery of nanoparticles to tumors exploits the leaky tumor vasculature and the impaired lymphatic drainage resulting in the retention of nanoparticles, a phenomenon known as the enhanced permeation and retention effect.^10^ This accumulation can be further enhanced through the active targeting approach, whereby nanoparticles functionalization with targeting molecules against a tumor antigen (e.g., antibodies, peptides, aptamers) is shown to improve nanoparticle uptake by the tumor.^11^

The human epidermal growth factor receptors 2 and 3 (HER2, HER3) are frequently overexpressed in brain metastases of several cancers, particularly HER2+ breast cancers.^12^ HER3 is an oncogenic receptor associated with drug resistance and poor prognosis,^13^ and we showed that HER3 is implicated in early brain metastasis.^14^ After receptor activation by its cognate ligand, neuregulin (NRG1), HER3 dimerizes with its preferred partner receptor, HER2, to transduce potent PI3K/Akt cell signaling that drives cell proliferation and apoptosis avoidance.^15,16^ The NRG1-abundant brain microenvironment may be implicated in the upregulation of HER3 and perpetual HER2-HER3 signaling, contributing to drug resistance in BM.^17^ Consequently, based on high expression frequency and pro-tumor activity in BM, HER3, and HER2 are therefore attractive therapeutic targets. Though several mAbs against HER2 or HER3 are available, single-targeting of the HER receptors in BM was not effective due to activation of compensatory HER-signaling when one of the HER receptors were being targeted.^18^ Therefore, utilizing HER receptors as targets for cytotoxic drug delivery may be a more effective approach than conventional receptor inhibitors that primarily aim to block the signaling pathway. With increasing importance of HER3 in cancers and brain metastases,^12,14,19^ we developed and investigated the potential of using a HER3-targeted nanomedicine for precise chemotherapy delivery.

In this proof-of-concept study, we synthesized and functionalized a pegylated hyperbranched polymeric nanoparticle (HBP) loaded with doxorubicin (DOX) via acid-labile hydrazone linkages. Subsequently, we conjugated the HBP with an HER3-targeting ligand, in the form of an anti-HER3/anti-PEG bispecific antibody fragment (bsAb), to enable tumor-targeting. The HER3-targeted HBP nanoparticle with DOX was then characterized and evaluated in vitro and in a breast cancer BM mouse model.

## 2 Experimental Section

### 2.1 Materials

Chemicals required for HBP synthesis were purchased from Sigma-Aldrich unless specified. These were filtered through a column of activated basic alumina to remove radical inhibitors before HBP synthesis. 4-Cyano-4-(2-phenylethanesulfanylthiocarbonyl) sulfanylpentanoic acid (PETTC) was synthesized as reported.^20^

### 2.2 HBP-DOX Synthesis

We utilized established materials as archetypical PEG-based nanomedicines as previously published and optimized in our laboratory.^21−23^

#### 2.2.1 HBP-DOX

Polymeric nanoparticles loaded with a DOX payload were synthesized using reversible addition–fragmentation chain-transfer (RAFT) polymerization as previously published in Janowicz et al.^21^ In short, poly(ethylene glycol) methacrylate (PEGMA) was utilized as the primary monomer component to impart solubility and biological compatibility. Ethylene glycol dimethacrylate (EGDMA) and Cyanine 5 methacrylamide (Cy5MA) were included at 6 and 0.6 mol % feed ratio relative to PEGMA as branching agents and fluorescent tags, respectively. The BOC-protected hydrazide methacrylate was also incorporated at 25 mol % relative to PEGMA for subsequent DOX loading steps, and polymerization utilized 4,4′-azobis (cyanovaleric acid) initiator (ACVA) as an initiator and 4-cyano-4-(phenylcarbonothioylthio)pentanoic acid as the RAFT agent. Following polymerization, the HBP was then modified to incorporate azido-chain end functionality, and the BOC protecting groups were removed through a trifluoracetic acid deprotection step. DOX was then incorporated under reflux conditions in methanol to incorporate DOX through a cleavable hydrazone linkage.

#### 2.2.2 HBP-DFO

For PET imaging applications, an equivalent HBP incorporating the chelator DFO to enable ^89^Zr-PET imaging was also synthesized as previously reported in Fletcher et al. and Mills et al.^22,23^ In brief, the synthetic approach followed that of HBP-DOX, incorporating PEGMA, EGDMA and Cy5MA. In this case, a mercaptothiazoline-functionalized RAFT agent was utilized to enable subsequent functionalization with an amine-functionalized deferoxamine (DFO) derivative to enable ^89^Zr chelation.

Both HBP materials were characterized as below, and a summary is presented in Table 1, demonstrating comparable physicochemical properties.

**Table 1:** Physical Properties of the DOX-Loaded Hyperbranched Polymer (HBP-DOX)

HBP	HBP molar mass^a^ (kDa)	arm size (kDa)^b^	arms per HBP	Cy5 per HBP^c^	DOX per HBP^c^
HBP-DOX	59	13.5	4.3	0.12	5.1
HBP-DFO	46	11.4	4.0	0.01	

a *M*_n_ (SEC-MALLS)

b *M*_n_ (NMR), estimated based on NMR integration of PEGMA relative to RAFT end groups

c Determined by UV–vis analysis

### 2.3 HBP Characterization

#### 2.3.1 Nuclear Magnetic Resonance (NMR)

All NMR experiments were conducted on a Bruker Avance 500 MHz high-resolution NMR spectrometer. Diffusion-weighted spectra (DOSY) were collected at a gradient strength (gpz6) of 50% for a minimum of 128 scans. Chemical shifts are reported as δ in parts per million (ppm) and referenced to the chemical shift of the residual solvent resonances (CDCl_3_^1^H: δ = 7.26 ppm; DMSO-*d*_6_^1^H: δ = 2.50 ppm). The resonance multiplicities are described as s (singlet), d (doublet), t (triplet), q (quartet), m (multiplet), or br (broad).

#### 2.3.2 Size Exclusion Chromatography (SEC)

SEC was conducted on a SEC-multiangle laser light scattering (MALLS) chromatographic system consisting of a 1515 isocratic pump (Waters), a 717 autosampler (Waters), Styragel HT 6 E and Styragel HT 3 columns (Waters), 2414 differential refractive index detector (Waters), and a Dawn Heleos laser light scattering detector (Wyatt). THF was used as the mobile phase throughout, with a flow rate of 1 mL/min. For effective light scattering analysis, the Cy5 fluorescence was first quenched by mixing 10 mg of polymer in 1 mL of THF along with a 4 mm^2^ piece of silver foil. The solution was bubbled with oxygen for 20 min and then left sealed under this high-oxygen atmosphere until no blue color remained (through overnight oxidation).

#### 2.3.3 Ultraviolet–Visible Spectroscopy (UV–Vis)

UV–vis was performed on a Nanodrop 2000C spectrophotometer (Thermo Scientific) using a quartz-glass pedestal with a path length of 1 mm path length. Absorbance maxima were recorded at 480 nm absorbance in triplicate for DOX and HBP-DOX, and concentrations of DOX per HBP were quantified relative to linear calibration curves of the DOX standards. HBP Cy5 fluorescence was also checked using UV–Vis at a 630 nm absorbance.

#### 2.3.4 pH-Dependent Doxorubicin Release Assay

The release profile of HBP-DOX was evaluated via a dialysis assay. Dialysis sacs (3.5 kDa SnakeSkin Dialysis Tubing; Thermofisher Scientific) containing 1 mL of HBP-DOX (1 mg/mL in PBS, pH 7.4) were floated in bottles containing PBS at pH of 7.4, 6.8, and 5.5 to mimic that of physiological, tumor microenvironment/early endosomes and late endosomes.^9^ Based on polymer drug loading (10%), 1 mg of HBP-DOX carries 0.1 mg of DOX. The setup was maintained at 37 °C with constant gentle shaking. PBS samples (1 mL) were taken from the external buffer of the dialysis sac at different time intervals and quantified spectrophotometrically on the Tecan Spark 10 M plate reader for doxorubicin content (excitation and emission wavelengths 495/590 nm). Four replicate measurements per time point were taken. Fluorescence measurements were baseline corrected to the earliest time point to determine the rate of drug release.

### 2.4 Bispecific Antibody (bsAb) Synthesis

For this proof-of-concept study, the HER3-targeting domain of bsAb corresponds to the antigen-binding domains of the clinically approved monoclonal antibody, lumretuzumab. Lumretuzumb is reported to compete and block neuregulin for HER3’s ligand binding domain.^24^ The amino acid sequences for the binding domains (Vh and V-kappa) of lumretuzumab (anti-HER3) were obtained from the “ImMunoGeneTics” database.^25^ The HER3 bsAb was produced in house (University of Queensland National Biologics Facility) as reported^22^ and is comprised of two single-chain variable regions (scFvs), one specific for HER3 and the other for methoxy polyethylene glycol (mPEG). Oligonucleotides encoding these were subcloned into a mammalian expression vector (pcDNA3.1), modified to include a C-terminal histidine tag for purification purposes, a mammalian leader sequence, a (Gly_4_Ser)_3_ linker between the two scFvs (α-HER3 and α-PEG) and codon optimization for expression in Expi293 human embryonic kidney cells (HEK, Thermo Fisher Scientific; Table S1). These vectors were designed in collaboration with the University of Queensland National Biologics Facility, with recombinant cloning outsourced to GeneArt. The amino acid sequence of an anti-PEG antibody was provided by University of Queensland National Biologics Facility.^26^ After expression in Expi293 cells, bsAb was purified through a series of size exclusion and affinity chromatography steps as previously reported. Purified α-HER3/α-PEG BsAb were sterile filtered via a 0.22 μm syringe filter after production, aliquoted in PBS and stored at −80 °C. Denaturing gel electrophoresis (SDS-PAGE) and surface plasmon resonance (Biacore T200) were used to assess HER3 bsAb purity and the binding kinetics between the bsAb and a recombinant HER3-Fc protein (348-RB, RnDSystems). To generate HER3-targeted HBP-DOX (HER3-HBP-DOX), the HER3 bsAb and HBP-DOX were combined in equimolar amounts for 1 h at room temperature to conjugate. We have previously showed that the approach of using α-*protein-of-choice*/α-PEG bsAb and the PEG-based HBP enables facile production of targeted polymeric nanomedicines.^21,22,27^

### 2.5 Cell Culture

Breast cancer cell lines, BT-474 (BT474) and MDA-MB-231 (MDA231), were sourced from the American Type Culture Collection and cultured in RPMI and DMEM supplemented with 10% fetal bovine serum (FBS) and antibiotic and antimycotic. BT474 cells were stably transduced with a luciferase expression cassette (pFB-Luc, Agilent) by lentiviral infection according to the manufacturer’s protocol. Lines were regularly tested to ensure that they were mycoplasma-free.

### 2.6 In Vitro Assays

#### 2.6.1 In Vitro Binding Assay

The binding assay was carried out using cell lines that express high (BT474) or low (MDA231) levels of the target receptor, HER3. These cells were cultured on glass coverslips to 70–80% confluence and then incubated with a low-serum (1%) medium containing 100 μg/mL of HER3-targeted HBP for 30 min. Then, cells were rinsed with phosphate-buffered saline (PBS) and fixed with 4% paraformaldehyde (PFA). To detect bound bsAb, blocking buffer (2.5% bovine serum albumin (BSA) in PBS) was applied for 30 min, then coverslips were incubated with an anti-his antibody (Abcam, 1:2000) overnight at 4 °C. A secondary goat anti-rabbit antibody labeled with Alexa Fluor 594 (1:400) was applied for 30 min at room temperature, and then coverslips were again washed with PBS, and mounted in ProLong Gold antifade reagent containing 4′,6-diamidino-2-phenylindole (DAPI; Thermofisher Scientific). Cells were imaged using an Olympus Axio fluorescent microscope at 20× magnification.

#### 2.6.2 In Vitro Antibody Competitive Binding Assay

BT474 cells were cultured on glass coverslips to 70–80% confluence and then incubated with 600 μL of PBS containing HER3-HBP (80 μg/mL) with or without scFvs based on the HER3 antibody, seribantumab (1.2 mg/mL). After 3 h of incubation at 4 °C, the cells were rinsed with PBS and fixed with 4% PFA coverslips and mounted in ProLong Gold antifade reagent containing DAPI (Thermofisher Scientific). Cells were imaged using the Zeiss LSM 710 confocal laser scanning microscope at 40× and analyzed using QuPath Bioimage Software^28^ for Cy5 intensity and cell positivity.

### 2.7 Cytotoxic Assay

BT474 cells were plated in 96-well plates (10,000 cells/well) in complete medium (RPMI with 10% FBS). The next day, the complete medium was replaced with different formulations of the treatment media: PBS, free DOX, HBP, HBP-DOX, HER3-HBP, or HER3-HBP-DOX at several concentrations prepared in RPMI with 1% FBS. Based on 10% polymer drug-loading, 1 mg of HBP-DOX is equivalent to 0.1 mg of doxorubicin. Each treatment was replicated across five wells. An equimolar ratio of HBP to bsAb (1:1) was utilized to generate receptor targeted constructs throughout the following studies as this has previously been demonstrated effective in producing enhanced tumor retention.^21,29^ After 48 h, cytotoxicity was assessed using the colorimetric MTT (3-(4,5-dimethylthiazol-2-yl)-2,5-diphenyltetrazolium bromide) assay. MTT substrate (1 mg/mL in complete medium) was added to each well, and the plate was incubated at 37 °C for 30 min. MTT (yellow compound) is converted to formazan (purple compound) by metabolically active cells. Thereafter, the medium was discarded, and wells were washed with PBS before 0.2% acidified isopropyl alcohol was added to each well to dissolve the formazan compound. Plates were placed on a shaker at 37 °C for 5 min, and the relative formazan content was determined spectrophotometrically (540 nm) using a Tecan Spark 10 M plate reader.

### 2.8 Brain Metastasis Xenograft Model

All studies were conducted within the guidelines of the Animal Ethics Committee of The University of Queensland, and the Australian Code for the Care and Use of Animals for Science Purposes (approvals: AIBN/CAI/530/15/ARC/NHMRC and UQCCR/186/19). Xenografts were established in 4 week old female NOD SCID mice (Australian Resources Centre). Mice were anesthetized with 2–4% isoflurane in oxygen at a flow rate of 1 L/min during engraftment, treatments, and in vivo imaging. Before internal carotid artery injection of cancer cells, fur on the neck region was shaved, and mice were secured under a dissection microscope in the supine position. The shaved area was disinfected using povidone-iodine antiseptic solution (betadine) and a surgical drape was laid over the mouse to isolate the surgical field from the nonsterile area. We generated the BM animal model by injecting 250,000 BT474 cells into the internal carotid artery of mice as described by us in Lim et al.^30^ Nine mice were xenografted for each treatment group, and those mice without detectable intracranial tumors or that failed to recover after xenografting were excluded from analysis.

### 2.9 In Vivo Imaging Studies

#### 2.9.1 Bioluminescence and Fluorescent Optical Imaging (BLI)

In vivo bioluminescence optical imaging (BLI) was used to evaluate disease progression and tumor burden (IVIS Spectrum imaging system). Mice were injected peritoneally with luciferin potassium salt in saline (VivoGlo, Promega) at 10 μL/g of body weight 15 min before imaging. Mice were then anesthetized with isoflurane 5 min before imaging and identical image acquisition settings and regions of interest were used to obtain total radiance values (Table S2).

The bioluminescence imaging (BLI) intensity raw data were plotted against time (weeks). Then, an exponential growth curve was fitted, and the growth rate constant (K) of the treatment groups was determined using GraphPad Prism software (v9.2).

#### 2.9.2 Positron-Emission Tomography-Magnetic Resonance Imaging (PET-MRI)

BM development was assessed by T2-weighted MRI as previously described.^21^ The mice were imaged 3 weeks after intracarotid artery injection of BT474 cancer cells to check for brain tumor formation and 3 weeks after their initial dose (week 7) to obtain post-treatment tumor volume. The mice were anaesthetized utilizing isoflurane (induced at 2–3% in oxygen and reduced to 1–2% as required) and placed in a Bruker 7T Small-Bore ClinScan MRI system (with the Siemens VB17 software; acquisition settings in (Table S2). 3DSlicer software (v4.10.1) was used for MR visualization, and the Segmentation module was used for estimation of brain tumor volume.^31^

For PET-MRI studies, HBP-DFO was labeled with ^89^Zr (supplied by PerkinElmer in 1 M Oxalic acid) as previously reported.^32,22^ BsAb was then added to PBS-buffered [^89^Zr]HBP-DFO and incubated for 45 min prior to injection to generate targeted constructs. Anaesthetized mice were then injected with 4–5 MBq of [^89^Zr]HBP-DFO (untargeted or HER3 targeted) via the tail vein. After 48 h, anaesthetized mice were placed in a Bruker 7T MRI system with a removable PET insert containing 3 rings of 16 detector blocks with 15 × 15 LSO crystals (1.6 × 1.6 × 10 mm) per block (Operating under Inveon Acquisition Workplace). A 60 min PET static emission scan was acquired simultaneously with MRI (settings in Table S2).

### 2.10 Treatments

Mice bearing intracranial tumors (confirmed via BLI and MRI) were treated 4 weeks after intracarotid injection. The treatment groups and the number of assessable mice per group were saline (*n* = 9), free DOX (*n* = 7), HBP-DOX (*n* = 8), and HER3-HBP-DOX (*n* = 7). Formulations were prepared in a background of PBS and administered via tail vein injection (equivalent to 3 mg/kg free DOX) twice weekly. Treatment continued until the conditions for euthanasia were reached (poor physical condition or weight loss >20%).

### 2.11 Ex Vivo Tissue Studies

#### 2.11.1 Tissue Collection

For ex vivo biodistribution imaging, organs were arranged on a Petri dish and imaged for Cy5 to detect the HBP distribution using the IVIS Spectrum system. Cy5 fluorescent imaging, emission and excitation filter were set at 670 and 600 nm, respectively. Images were processed using the Living Image Software (IVIS Spectrum, PerkinElmer). Transcardiac perfusion with heparin followed by 4% paraformaldehyde (PFA; 1 mL/min) was performed to preserve the histology of the brain after necropsy. Tissues were fixed in 4% PFA, then processed as formalin-fixed paraffin-embedded (FFPE) blocks for immunohistochemistry (IHC).

#### 2.11.2 Histology and Immunohistochemistry

Tissue sections (4 μm thick) were mounted on charged slides (Superfrost Plus, Thermo Scientific) and dried overnight at 38 °C. For IHC, the sections were first rehydrated in a series of xylene and ethanol, and then heat-induced antigen retrieval was performed in a pressurized chamber (Biocare Medical). The sections were heated to 95 °C for 30 min in sodium citrate buffer (pH 6.5), then 3% hydrogen peroxide was applied to quench endogenous peroxidase activity, and the slides were blocked in Sniper Blocking Solution (Biocare Medical) for 30 min.

The primary antibody against a germ cell marker, mouse vasa homologue protein (MVH) (Polyclonal, Abcam, ab13840), was diluted in Da Vinci Green (Biocare Medical) at 1:2000 and applied to the sections and incubated overnight at 4 °C in a humidified slide chamber. Sections were washed in Tris-buffered saline (TBS; 3 × 5 min) before incubating with secondary antibody. A horseradish peroxidase secondary was applied for 30 min before rinsing with TBS (3 × 5 min). Sections were developed with DAB substrate solution (Biocare Medical) for 5 min, then rinsed in water, counterstained in hematoxylin, and coverslipped using DPX mountant (Sigma-Aldrich). Slides were scanned by using the Aperio Turbo digital slide scanner (Leica). Images were analyzed by using QuPath software.

### 2.12 Statistical Analysis

Statistical analysis was performed using GraphPad Prism (v9.2), with individual statistical tests indicated in the figure legends. All bar graphs indicate the mean and standard error of the mean.

## 3 Results

### 3.1 Generation of Polymeric Nanomedicines

The hyperbranched polymer (HBP) was produced as an archetypical polymeric drug carrier platform,^21−23^ and composed primarily of polyethylene glycol. It measures 5–10 nm in size and has well-reported pharmacodynamic properties and long circulation (>12 h half-life in blood).^32,33^ We synthesized the HBP via a previously established RAFT polymerization protocol using PEGMA as monomer and EGDMA to provide the branch points between chains.^21,34^ Doxorubicin (DOX) was attached to HBP via a hydrazone linker for acid-responsive drug release. Cy5 fluorophore was attached to enable ex vivo biodistribution analysis via Cy5 fluorescence. The synthetic scheme is provided in (Figure S1). NMR spectroscopy was conducted to confirm the incorporation of RAFT agent end-group, as well as the PEGMA, Cy5-MA, and EGDMA monomeric units. NMR also confirmed the removal of the TBMC BOC group following deprotection of the amine moieties prior to post modification with DOX (Figures S2–S3). Successful conjugation of DOX to HBP via the hydrazone bond was confirmed by the peaks between 7.75 to 8.0 ppm (Figure S4). UV–vis spectroscopy of HBP-DOX confirmed the attachment of DOX and Cy5, with retention of absorbances at 480 and 650 nm following purification, respectively (Figure S5). Overall, the molecular weight of HBP-DOX is about 59 kDa with 4 branches per polymer. The Cy5 and DOX were conjugated at 0.12 and 5.1 per polymer, equivalent to a drug loading of 5% w/w (Table 1). The produced and characterized HBP-DOX was then lyophilized to a dry oil, stored at −20 °C, and subsequently dissolved immediately before each experiment.

We used different conjugation methods for component attachment in the HBP delivery system. The Cy5 fluorophore is conjugated via stable amide bonds, while DOX is attached through hydrolysis-prone hydrazone bonds. From experience, this design strategy ensures the Cy5 fluorophore remains intact during the delivery process while enabling controlled drug release at acidic tissue sites.^34,29,27,35,21^

The acid-sensitive hydrazone linkage exploits the natural variation in pH between healthy (pH7.4) and tumor tissue (pH 6.8) and the endosome compartment (pH 4.5–6.8) to achieve controlled drug release. To confirm selective drug release under desired pH conditions, we evaluated DOX release from the HBP semiqualitatively by dialyzing HBP-DOX with or without HER3 bsAb (HER3-HBP-DOX) in PBS buffers with specific pH levels. The trend of DOX released is determined by periodically measuring the fluorescence intensity of doxorubicin in the external buffer by spectrophotometry periodically.

**Figure 1:**
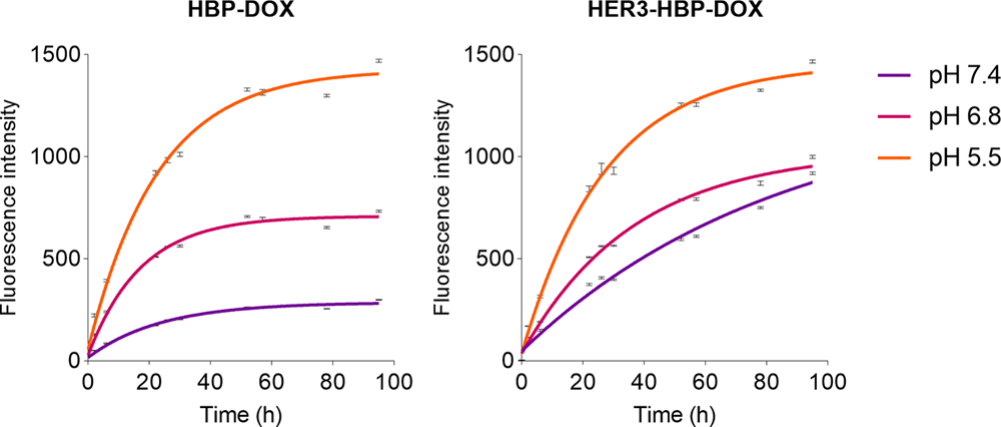
HBP-DOX drug release. Graph shows the increasing fluorescence intensity of doxorubicin, illustrating the pH-dependent release of doxorubicin from HBP-DOX with or without HER3 bsAb conjugation, observed over 96 h at 37 °C in PBS. The pH values of 5.5, 6.8, and 7.4, represent acidity levels of endosomal compartments, tumor tissue microenvironment and normal tissue, respectively. Mean ± standard error shown. The *Y*-axis represents fluorescence intensity of doxorubicin at emission wavelength of 590 nm.

The results showed that in both groups, HBP-DOX and HER3-HBP-DOX, DOX fluorescence intensity was gradually detected in the external buffers over time in all the pH conditions. The DOX release was largely completed by the DOX intensity levels plateauing after 4 days. In both groups, we observed a similar trend showing the greatest intensity detected at pH 5.5 and the lowest level detected at pH 7.4 (Figure 1). The difference in size and shape between the untargeted and targeted-HBP nanoparticles was expected to cause slight variation to their drug-release profiles.^36^ Nonetheless, this assay shows that the drug-release from HBP-DOX with or without a targeting ligand was gradual and controlled. In contrast, when free DOX was dialyzed, DOX intensity levels detected in the external buffer peaked rapidly within 12 h (Figure S6). This is expected, as the immediate release profile is typical for doxorubicin administered in its free form (not bound to a carrier). This experiment confirmed that HBP-DOX is stable at physiological pH and capable of sustained controlled drug release at acidic pH resembling peritumoral and endosomal conditions.

For the subsequent PET experiment, we synthesized an HBP incorporating the chelator DFO instead of the therapeutic DOX to enable ^89^Zr binding. The resulting [89Zr]HBP-DFO had physicochemical properties (Table 1) comparable to those of HBP-DOX and allows quantitative preclinical imaging to further probe biodistribution in the brain.

### 3.2 Characterization of the α-HER3/α-PEG Bispecific-antibody

**Figure 2:**
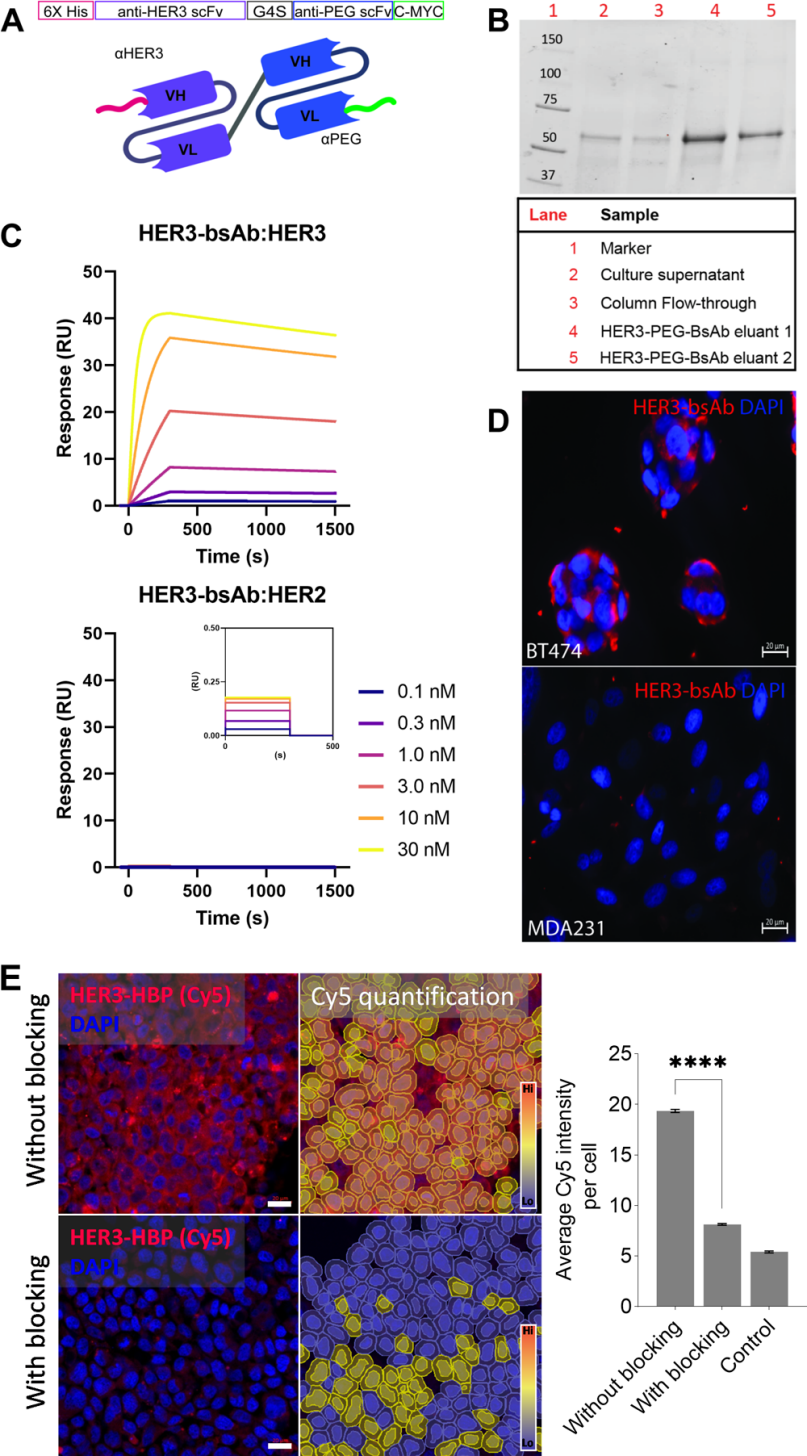
Characterization of purified HER3 BsAb. (A) BsAb gene design and format consisting of a HER3 binding domain (purple) and a PEG binding domain (blue) joined by a glycine-serine linker (G_4_S). Six histidine (6× Hist) and C-MYC tags are designed for protein purification and detection. Illustrations of a BsAb on a PEG-based hyperbranched polymer. (See Table S1 for full amino acid sequences). (B) Coomassie-blue-stained SDS-PAGE gels loaded with HER3-PEG-BsAb. Molecular mass markers are indicated (kDa). Column eluants (lanes 4 and 5) showed bands corresponding to the expected size of ∼55 kDa. (C) Multicycle kinetic sensorgrams for HER3 bsAb. Plots show association and dissociation phases for increasing concentration of analytes, HER3 extracellular domains at 0, 0.3, 1, 3, 10, and 30 nM (Top plot). There was a very low level of detection when HER3 bsAb was assayed against unmatched HER2 extracellular domain (lower plot, inset) confirming selective receptor binding. (D) HER3-bsAb in vitro binding in BT474 (HER3+) and MDA-MB-231 (HER3−). (E) Cy5 labeled HER3-HBP in BT474 with or without second HER3 antibody blocking. Average Cy5 intensity per cell was quantified and plotted. (Unpaired *t* test: ****, *p* ≤ 0.0001). Cells imaged at 20× magnification using immunofluorescent microscopy. Scale bars represent 20 μm.

Bispecific antibody (bsAb) was produced to facilitate targeting of the DOX-loaded HBP. This engineered bsAb comprises two scFvs joined by a flexible linker. One binding domain targets a disease marker of interest, HER3, while the other end targets the methoxyPEG backbone of the HBP^26^ (Figure 2A). We have previously used this approach as a simple method for developing targeted nanomedicines through formation of noncovalent complexes of HBP and targeting ligand.^21,22,27,35,37^

Western blot analysis of the HER3 bsAb showed single bands at the expected size of 55 kDa, demonstrating successful expression of the bsAb product with limited degradation (Figure 2B).

Surface plasmon resonance was used to characterize the binding affinity between the bsAb and the target (recombinant proteins of the HER3 extracellular domain). The results demonstrated a strong antigen binding affinity of HER3 bsAb against the extracellular portion of human HER3 recombinant protein, with an equilibrium dissociation constant (KD) of 1.42 × 10^–10^ M (Figure 2C). The binding affinity was comparable to published binding affinity of parental antibody, lumretuzumab, 5.0 × 10^–10^ M.^24,38^ The association rate (*K*_a_, 1/Ms) and dissociation rate (*K*_d_, 1/s) of HER3 bsAb are 7.13 × 10^5^ and 1.01 × 10^–4^. To check for nonspecific binding, we exposed HER2-coated sensor chip with HER3 bsAb. The result shows that no binding was detected using a sensor chip coated with negative control substrate (i.e., no binding detected between HER3 bsAb and a HER2-coated chip) (Figure 2C).

Lastly, we confirmed the specificity of HER3-targeting via two in vitro binding assays. In the first assay, we incubated the HER3-bsAb with breast cancer cell lines that express high (BT474) or low levels of HER3 (MDA231). Expression of HER3 was verified by Western blotting (Figure S7). Using immunofluorescent microscopy, we detected HER3 bsAbs in BT474 (HER3 positive cell line) and not in MDA231 (HER3 negative cell line) (Figure 2D).

We performed an antibody–antigen competition binding assay, where HER3-HBP with or without a HER3 bsAb was added to BT474. The HER3-HBP was generated by incubating Cy5-labeled HBPs at a 1:1 molar ratio with HER3 bsAb. The competing HER3-antibody is an scFv antibody based on the clinical HER3 antibody, seribantumab,^39^ which competes with HER3-HBP for HER3’s neuregulin ligand binding domain. We observed that there were significantly more Cy5 positive cells in the sample without competitive HER3-antibody blocking than in the sample with blocking (Figure 2E). Together, the results demonstrated that the HER3-targeted HBP were specific and bound to the intended native HER3 receptors on the cell surface. The experiment also demonstrated the success and ease of functionalizing HBP with HER3/PEG bsAb. Based on the HBP to bsAb molar ratio (1:1), the targeted HBP will be approximately 115 kDa. We conjugate equimolar of HBP to bsAb to generate HER3-targeted HBP throughout this study as this ratio has been demonstrated to be effective in enhanced tumor retention.

### 3.3 Cytotoxicity of HBP-DOX to BT474 Breast Cancer Cells

The cytotoxicity of the nanoparticles was investigated by treating BT474 cancer cells for 48 h at a range of concentrations followed by performing an MTT assay to determine cell viability. The result was presented as a percentage fold change relative to that of the untreated (PBS) control group.

**Figure 3:**
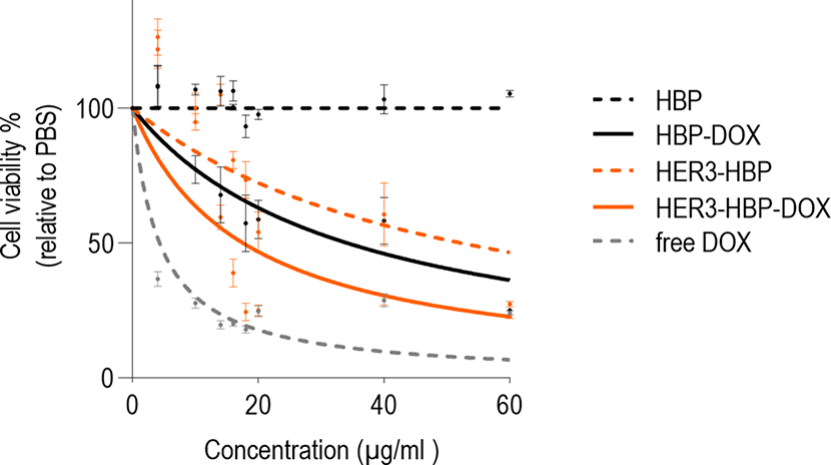
Cytotoxicity of HBP-DOX on BT474. Nonlinear regression plot shows cell viability of BT474 after 48 h treatment with free DOX, or the HBP formulations. The *x*-axis indicates concentration, and the *y*-axis shows cell viability relative to an untreated (PBS) control. Data shown are the means ± standard error of the mean of three independent experiments.

Free DOX-treated cells exhibited the lowest cell viability, whereas cells treated with untargeted HBP without DOX-loading were comparable to the untreated control group suggesting that HBP alone, without targeting bsAb or DOX payload, is not cytotoxic (Figure 3).

Next, we investigated the effect of targeting HER3 using HBP functionalized with a bispecific antibody (bsAb) on BT474 cell viability. Our findings demonstrated that the HER3-HBP exhibited greater cytotoxicity than untargeted HBP (Figure 3), likely through inhibiting the HER2-HER3 proliferation and survival pathway.

Last, we investigated if the addition of a chemotherapeutic payload (DOX) to HBP enhances cytotoxicity. We incubated equimolar amounts of HBP-DOX and HER3 bsAb to generate HER3-HBP-DOX. We observed that HBP with the DOX payload (HBP-DOX) displayed greater cancer cell killing relative to HBP alone. Similarly, HER3-HBP-DOX exerted greater cytotoxicity than HER3-HBP (Figure 3). The greatest cytotoxicity was seen in HER3-HBP-DOX. We noted at low drug concentration (<20 μg/mL), some samples exhibited greater MTT reading than PBS-treated cells. This suggests heighten cellular metabolism and growth at low noncytotoxic HBP-DOX exposure. The half maximal inhibitory concentrations (IC50) are 34.2, 52.3, and 17.6 μg/mL for HBP-DOX, HER3-HBP, and HER3-HBP-DOX, respectively, and the data are shown in Figure S8.

### 3.4 Biodistribution of HBP in Healthy Animals

**Figure 4:**
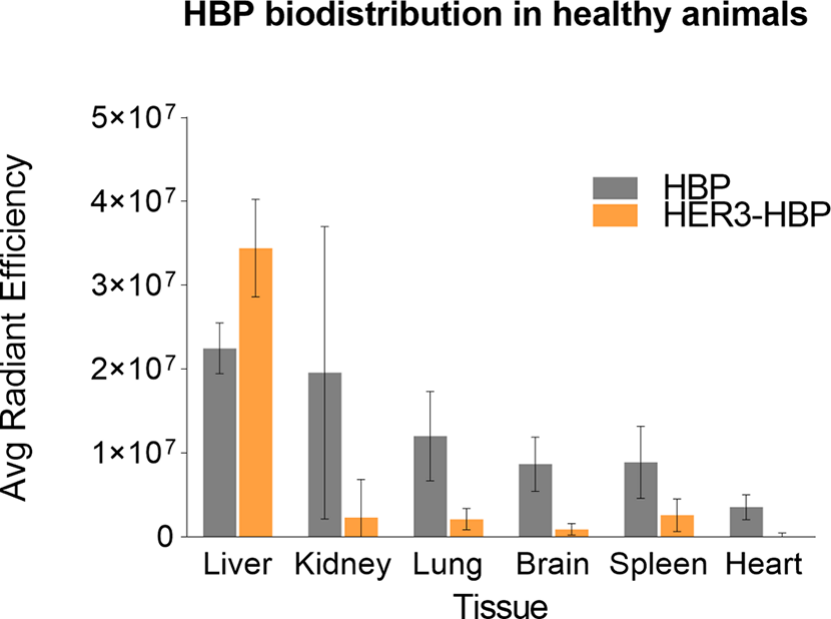
HBP biodistribution in healthy animals. Graph illustrates accumulation of untargeted (HBP) and targeted formulations (HER3-HBP) in healthy organs based on ex vivo fluorescent imaging and measurement of Cy5, compared to saline-injected controls (*n* = 3 per group). Radiance was normalized to saline-injected mice, and the background subtracted.

We next investigated the biodistribution of HBP by administering untargeted-HBP, HER3-targeted HBP in healthy mice followed by assessing Cy5 (incorporated within the HBP) fluorescence intensity using ex vivo organ imaging. The organ with the highest Cy5 intensity was the liver, suggesting that HBP is primarily cleared via the hepatic route (Figure 4).

We also noted stronger Cy5 intensities in livers from mice treated with HER3-HBPs compared to untargeted-HBP. We propose that the functionalization of low-fouling PEG-based HBP with proteinaceous targeting ligands (bsAb) likely resulted in their enhance interactions with components of the mononuclear phagocytic system driving more rapid (hepatic) clearance from the circulation.^22,40^ For brain biodistribution, the conjugation of HER3 bsAb to HBP results in lower accumulation in the brain compared to that of untargeted HBPs alone. This outcome is anticipated due to the increased size of the targeted HBPs, which are around twice as large as the untargeted HBPs (approximately 115 kDa vs 55 kDa). Consequently, in normal animals with an intact and functional blood–brain barrier (BBB), larger molecular materials exhibit reduced passage across the BBB. This observation implies that in surrounding healthy brain tissues with intact BBB, there would be a decreased accumulation of nanomedicine minimizing the potential for nonspecific drug toxicity. While trend differences in Cy5 fluorescence intensity were noted, these differences did not reach statistical significance under the conditions of our study.

### 3.5 In Vivo Treatment Effects

**Figure 5:**
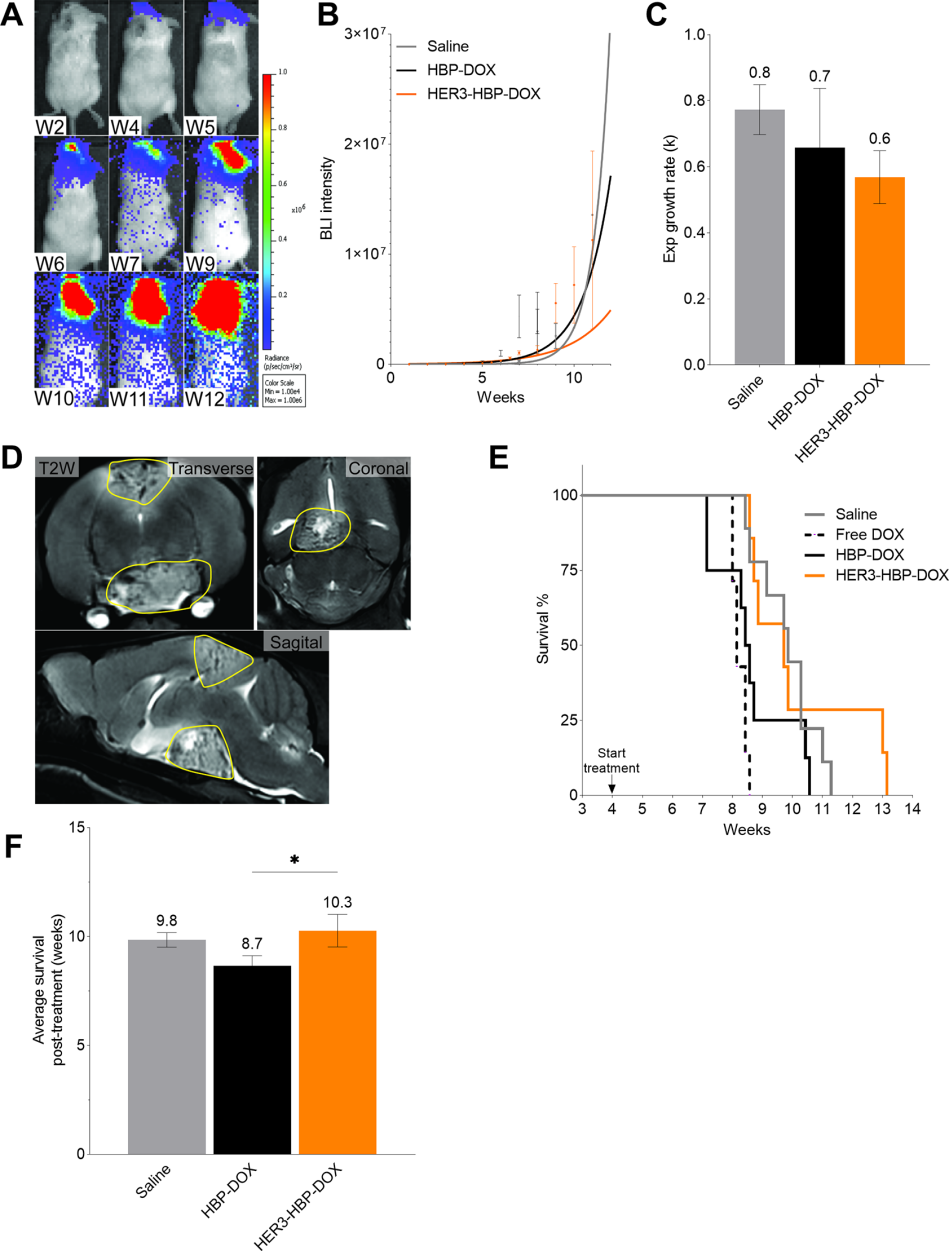
Effect of treatment on BM progression and survival. (A) Representative illustration of bioluminescence imaging (BLI) of a mouse with developing BM 2 weeks (W) after injecting cancer cells. Standardized regions of interest were drawn on the head of the animals, and the bioluminescence signals were measured (B) Exponential growth curves were fitted to the BLI data and (C) growth rates (*k*) were plotted. Relative to saline group, the HER3-HBP-DOX treatment reduced tumor growth. (D) T2-weighted MR images of a mouse with BMs in the cerebral cortex and hypothalamus region (circled yellow). (E) Kaplan–Meier analysis of HBP-treated BT474 BM-bearing mice. Treatment commenced at week 4 and consisted of saline, free DOX, HBP-DOX, and HER3-HBP-DOX (*n* = 9, 7, 8, 7). (F) Comparison of the average survival of treatment groups. Data shown means ± standard error. (ANOVA test; *, *p* < 0.01).

We assessed therapeutic efficacy of HBP nanomedicine by comparing impact on intracranial tumor progression using bioluminescent signals and BM survival duration. We generated BM mouse models by injecting luciferase-tagged BT474 into the internal carotid artery of mice.^30^ Intracranial tumors were monitored weekly by measuring luciferin–luciferase bioluminescence intensity from the heads of the mice using bioluminescence optical imaging (BLI) (Figure 5A). The presence of intracranial tumors was confirmed by observation of bioluminescence signals localizing to the head of the animals at 4 weeks postengraftment. The mice were then dosed twice weekly, and their physical conditions were monitored closely. The treatment groups were saline (*n* = 9), free doxorubicin (DOX; *n* = 9), untargeted HBP-DOX (*n* = 8), and targeted HER3-HBP-DOX (*n* = 7). All mice were dosed at equivalent to 3 mg/kg DOX in appropriate formulations. Of note, animals from the free DOX group were euthanized after 4 weeks of dosing (at week 8) due to severe physical deterioration (e.g., weight loss of >20%) associated with systemic toxicity. As the number of data points from free DOX group were insufficient for meaningful analysis, data points were excluded.

#### 3.5.1 BM Progression

In the remaining treatment groups, we observed increasing bioluminescent signals from the animals’ head region, and these signals remained throughout the study, suggesting persistent brain tumors (Figure 5A). We generated exponential growth curves and determined rate constant (*k*) (Figure 5B) to evaluate the differences in growth trends among the groups. The result showed that throughout the study, the HER3-HBP-DOX group had the lowest BLI intensity and rate constant (*k*) compared to saline and HBP-DOX groups, suggesting that HER3-HBP-DOX exerts an inhibitory effect on tumor growth (Figure 5B,C). We noted that this difference is not statistically significance. To confirm the presence of intracranial tumors, we conducted in vivo assessment of the BM tumors and determined their volume via T2-weighted MRI at week 7, following 3 weeks of treatment (Figure 5D). From the MRI, all of the animals were observed to have intracranial tumors with varying sizes. We noted that the HER3-HBP-DOX group had smaller brain tumors relative to saline and HBP-DOX groups at the week 7 time point (ANOVA test, 0.703 and 0.018 respectively; Figure S9).

#### 3.5.2 Survival

The effect of the treatment on animal survival was assessed by Kaplan–Meier analysis. Among the groups, HER3-HBP-DOX treated mice showed the longest survival duration (Figures 5F and S9). We noted that this did not attain statistical significance when compared to the Saline group. Next, we found that the HBP-DOX-treatment of mice did not improve survival and the mice had shorter survival time compared to saline group (Figures 5F and S9). This is expected considering that tumors from the HBP-DOX group are among the largest. Notably, both nanomedicine groups (HBP-DOX and HER3-HBP-DOX) exhibited better survival than free DOX-treated mice, suggesting that the nanomedicine-formulations are better tolerated than free DOX. Overall, this experiment showed that HER3-HBP-DOX has the potential to reduce tumor growth and size while extending survival duration. On the other hand, untargeted HBP-DOX did not confer any therapeutic benefit. As HBP-DOX and HER3-HBP-DOX dosages were designed to match the dosing limit of free DOX at 3 mg/kg, the result suggested that we may be underdosing with the nanomedicine formulation.

### 3.6 Understanding Nanoparticle Biodistribution in BM Using PET/MR and Cy5 Fluorescent Imaging

To provide additional insight into the therapeutic study findings, we utilized molecular imaging approaches to probe nanomaterial localization and delivery in our BM model.

#### 3.6.1 [^89^Zr]-PET Imaging of HBP

**Figure 6:**
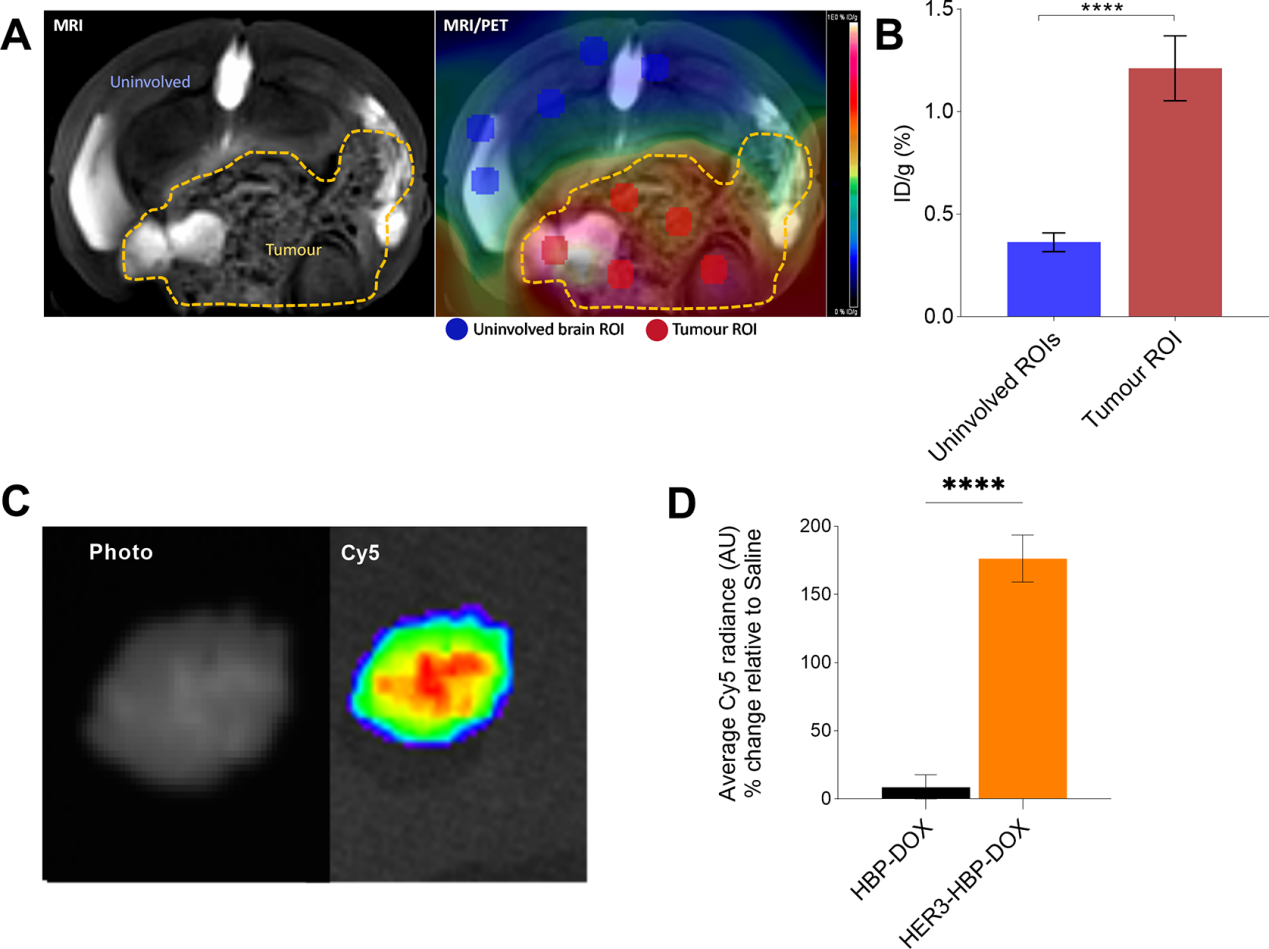
Biodistribution of HBP in brain metastasis via radiolabeled HBP and Cy5-labeled HBP. (A) Representative T2-weight MRI (coronal view) depicting brain tumor (yellow dotted region) and uninvolved brain region from a mouse injected with ^89^Zr-HER3-HBP. Superimposing PET overlay on MRI image demonstrated that the tumors have higher uptake of the nanomedicine (white, red, yellow) compared to the uninvolved regions (blue/green). Regions of interest (5 each) from the uninvolved brain and tumor were selected from each animal. (B) In all treatment groups, there was increased radioactivity detected from region of interests of tumor regions (*n* = 30 ROIs) compared to uninvolved brain regions (*n* = 30 ROIs) (1.21 vs 0.36%ID/g) (combined groups). (C) Representative photograph of an excised whole brain (left) and with Cy5 fluorescent overlay (Right). (D) Graph illustrates HBP accumulation in excised brains based on ex vivo bioluminescent imaging. Highest Cy5 intensity was observed in the HER3-HBP-DOX treated group. Data are normalized as percentage change relative to the saline group. Means ± standard error. (ANOVA test: ****, *p* ≤ 0.0001)

Quantitative preclinical PET imaging was utilized to further probe the accessibility of the polymers to the developing tumors. An archetypical HBP capable of binding ^89^Zr radioisotope with the chelator DFO (HBP-DFO) for imaging was synthesized (Table 1). HBP-DFO was labeled with ^89^Zr in-house, and at week 7 postengraftment when tumors were well established, three mice from each treatment group were selected and administered [^89^Zr]-HBP or [^89^Zr]-HER3-HBP (*n* = 3 mice/group). After 24 h, positron emission tomography (PET)/MR imaging was performed. Brain tumors were visualized as hyper-intense regions on T2-weighted MRI (Figure 6A). From each animal, we selected the MRI coronal slice that displayed the largest dimension of the tumor, and on its corresponding PET data, five regions of interest were annotated in areas of uninvolved brain and tumor (Figure 6A). The injected dose per gram (ID/g) derived from these ROIs were grouped into uninvolved brain and tumor regions and compared (*n* = 60 ROIs each). In all treatment groups, the localized concentration of ^89^Zr-labeled HBP in tumor associated regions of the brain is 1.25%ID/g compared to uninvolved brain region (0.31% ID/g) (Figure 6B), indicating preferential localization of HBP nanoparticles in tumor regions. Comparative analyses were not performed between the groups due to only one slice of the MRI and PET data being assessed.

#### 3.6.2 Ex Vivo Imaging of Fluorophore-Labeled HBP

To further investigate tumoral localization, we performed Cy5 fluorescent imaging of brains harvested from the animals after the end point (Figure 6C). We found localized Cy5 signals in the brains of HBP-DOX and HER3-HBP-DOX relative to saline treated animals (Figure 6D). Comparing Cy5 intensity among the nanomedicine treated mice, we found that Cy5 intensity was highest in the brains of HER3-HBP-DOX treated mice (14-fold greater than untargeted) (Figure 6D). This shows that active targeting of HBP-DOX achieved greater nanoparticle retention than passive delivery of HBP-DOX. This supports preclinical PET imaging results and provides more nuanced insight into the influence of specific receptor targeting.

These experiments show that HBP localized within metastatic brain tumor regions and that receptor targeting with bsAb functionalization improved the retention of nanoparticles in a target-dependent manner.

### 3.7 Off-Target Effects of HBP Nanomedicine

While tumor accumulation and therapeutic efficacy are the key focus, the off-target effects of interventions and resulting dose limitations due to tolerability concerns are also important factors in patient outcomes. To probe this, we examined the off-target effects of HBP-DOX on the general health status, heart, and fertility relative to that of free doxorubicin.

#### 3.7.1 Body Weight

We analyzed the change in mice bodyweight as an indicator of animal health.^41^

**Figure 7:**
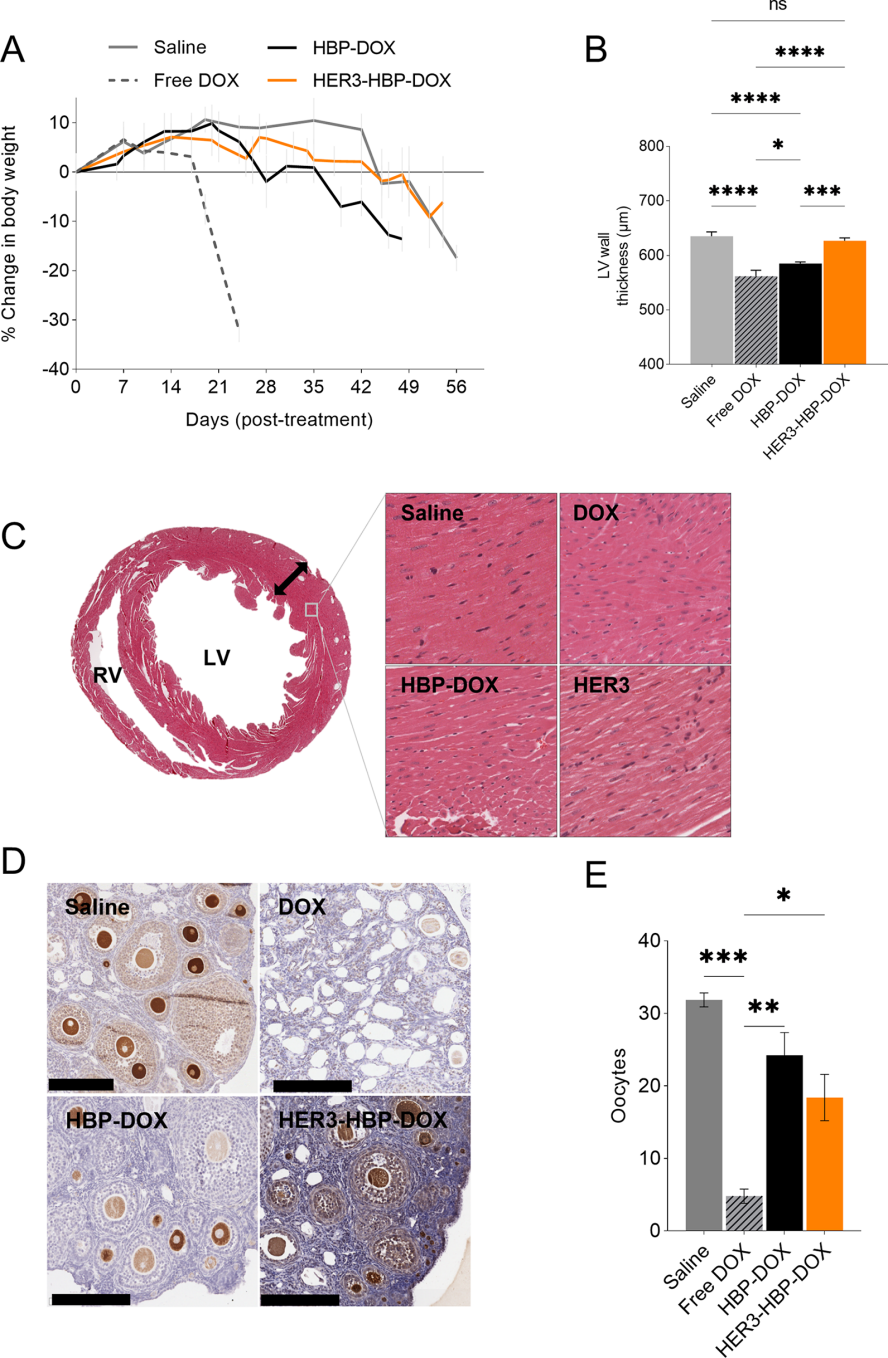
Effect of HBP nanomedicine on body weight, left ventricular wall and ovarian reserve. (A) Mice were weighed weekly, and the graph shows percentage change in weight after the first dose. (B) Twenty left ventricular wall (LV) measurements were taken along the left myocardium, and bar graph shows the left ventricular wall thickness of mice from different treatment groups: saline (*n* = 8), DOX (*n* = 8), HBP-DOX (*n* = 4), and HER3-HBP-DOX (*n* = 5). (C) Representative midventricular cross-section of a murine heart showing left and right ventricles (LV, RV). Black arrow denotes measurements taken along the left myocardium. Panel shows representative micrographs of myocardiums from the treatment groups. Scale bars represent 100 μm. (D) Representative of MVH-stained oocytes (brown) in the ovaries from different treatment groups. White spaces were prevalent in the ovaries of mice treated with free doxorubicin, whereas more oocytes were observed in the nanomedicine treated groups. Scale bar represents 200 μm. (E) Graph comparing the number of oocytes per ovary in the different groups. Data shown are means ± standard error of mean (ANOVA test: *, *p* ≤ 0.1, **, *p* ≤ 0.01, ***, *p* ≤ 0.001, ****, *p* ≤ 0.0001).

Saline-treated mice maintained their body weights until day 42 before they succumbed to BM (Figure 7A). Notably, free DOX-treated mice experienced rapid weight loss and physical deterioration after 7 days post-treatment. This is in line with accepted response in animals for conventional chemotherapy which imparts systemic toxicity.^42^ In comparison, mice treated with HBP nanomedicines maintained their body weights for longer periods before succumbing to a progressive disease burden. With fewer impacts on the health, animals treated with HB-nanomedicines received more treatment doses over the course of this study. This is consistent with in vitro data showing that HBP and HBP-DOX are less cytotoxic compared to free drug, DOX (Figure 3).

#### 3.7.2 Cardiotoxicity

Chemotherapy-related cardiotoxicity is a dose-limiting factor which impacts treatment response and quality of life.^43^ Doxorubicin can induce dilated cardiomyopathy and the thinning of the left ventricular wall (LVW), leading to functional insufficiency and heart failure.^44^ To probe the impact of nanomedicine formulation, we compared the LVW thickness of treated mice (20 measurements/mouse) and myocardial morphology was analyzed by H&E staining (Figure 7B,C). We found that free DOX and HBP-DOX-treated mice have thinner LVW than saline-treated mice (Figure 7B). On the other hand, the LVW of HER3-HBP-DOX-treated mice was comparable to that of saline-treated mice. This showed that HER3-HBP-DOX was associated with less cardiotoxicity than free DOX and HBP-DOX.

#### 3.7.3 Genotoxicity

Systemic chemotherapy is often associated with toxicity toward the reproductive system, and we proposed that the nanomedicine formulation may mitigate this impact. We investigated the effect of the nanomedicine on the oocyte pool of female mice. Ovaries from the treated mice were immuno-stained for Mouse Vasa Homologue (MVH), a protein critical for the processing of primordial germ cells and highly expressed by follicles and oocytes.^45^ The stained oocytes were then enumerated (Figure 7E). In the saline group, numerous oocytes with strong MVH staining were observed (average 31 oocytes/ovary; Figure 7D). The ovaries of mice treated with free DOX consisted mostly of “empty pockets” with very few MVH stained structures resembling oocytes despite being treated with fewer doses than HBP-groups (average 5 oocytes/ovary: Figure 7D). Compared to DOX-treated mice, there were significantly more oocytes in HBP-DOX and HER3-HBP-DOX groups (6- and 4.5-fold more, respectively) (DOX vs all HBP-DOX, *p* = 0.0008), with visibly fewer “empty pockets” in the ovaries (Figure 7E). This result shows that HBP-based nanomedicines are less gonadotoxic than doxorubicin and may therefore be favorable for managing patients of child-bearing age.

## 4 Discussion

There is a lack of targeted therapeutic options for managing brain metastasis. There is only one U.S. Food and Drug Administration (FDA)-approved systemic therapy indicated for breast cancer BM:^46^ tucatinib with trastuzumab and capecitabine for patients with metastatic HER2+ breast cancer.^47,48^ This is partly due to the challenges associated with the dosing and scheduling of cytotoxic drugs, as they often result in adverse toxicity. Consequently, there is a major unmet clinical need for safe and effective therapies that can improve the clinical outcomes of BM patients. Therefore, in this study, we investigated the potential of using a targeted nanomedicine for delivering chemotherapy in BM.

Using a mouse model of brain metastasis, we evaluated the use of HBP for delivering doxorubicin through either passive or active targeting to HER3. Our findings showed that the HER3-HBP-DOX nanoparticles provided modest survival benefit over saline, free DOX, and untargeted HBP-DOX. The mice treated with HER3-HBP-DOX showed reduced tumor growth rates and smaller tumor volumes, indicating the inhibitory effect HER3-HBP-DOX has against BM tumor. It is noteworthy that despite using a lower doxorubicin dose (3 mg/kg) than published studies of maximum tolerated doses (7.5 mg/kg),^49^ the mice treated with free DOX experienced severe adverse chemotherapy effects such as acute weight loss, ruffled fur, lethargy, and labored breathing after 2 weeks of treatment. In contrast, the nanomedicine-treated mice, including HBP-DOX and HER3-HBP-DOX, did not manifest any signs of acute-treatment-associated toxicity and experienced longer survival. Exemplifying this, the nanomedicine-treated mice recovered and resumed their activity after each dosing, allowing for more treatments compared with free DOX treated mice.

We observed a limitation in using the luciferase-based measurement for intracranial progression. As luciferase-based bioluminescence is generated from the cellular metabolism of luciferin by tumor cells, bioluminescence intensity is not a definitive measurement for tumor size. Given that DOX was shown to disrupt mitochondria respiratory function,^50^ we postulated that the small tumors in saline group were more catalytically active, and hence brighter, compared to tumors in DOX-containing treatments. This would need further investigation in a dedicated experiment.

PET/MRI and fluorescence imaging were utilized to investigate the distribution of the nanomedicines in the brain in vivo and ex vivo. The results demonstrated the localization of both untargeted and HER3-targeted HBP-DOX in brain tumors region relative to uninvolved brain region. Additionally, the ex vivo fluorescent brain imaging revealed that HER3-targeted HBP-DOX has 14-fold more intracranial accumulation than untargeted HBP-DOX highlighting the advantage of the active targeting approach in enhancing drug delivery to brain tumors. These imaging experiments showed that the modest therapeutic performance of HER3-HBP-DOX was not due to a lack of intracranial drug accumulation.

The modest benefit in tumor response may be due to several compounding factors. First, using a targeting ligand with strong affinity could potentially reduce tumor penetration and limit the diffusion of nanomedicine in extracellular spaces,^51^ a phenomenon known as the “binding site barrier”.^51,52^ This phenomenon in combination with other physiologic barriers present in brain tumors, such as interstitial pressure, incomplete BBB permeability, and necrosis, could further reduced drug uptake and therapeutic inefficiency.^53^

Second, intracranial tumor cells may be exposed to sublethal concentration of DOX^49^ which could lead to upregulation of drug efflux mechanisms^54^ or DNA damage repair pathways^55,56^ resulting in survival and chemoresistance.^57^ The larger tumors observed in HBP-DOX treated mice raised the possibility that refractory pathways may be implicated in promoting tumor growth.

The NRG1-HER3-PI3K signaling pathway has been identified to play a role in promoting brain metastasis outgrowth and therapy resistance, making the targeting of HER3 in combination with a standard systemic drug a logical approach.^17^ However, it is possible that the HER3-targeted nanomedicine employed here may not have adequately suppressed HER3-PI3K signaling in tumor regions that have poor drug access. Previous studies have shown the prevalence of HER2-HER3 signaling dimers HER2+ BM and, in a neuregulin-abundant microenvironment, targeting HER2 alone with anti-HER2 clinical antibodies such as trastuzumab and/or pertuzumab did not abrogate HER2-HER3 dimerization. Similar challenges may arise for single-HER3 targeting. Additionally, the association between neuregulin and the HER2-HER3 dimer is stronger than between our HER3 bsAb for HER3 (equilibrium dissociation constants (*K*_D_) 0.02 vs 0.142 nM, respectively).^16^ Hence, it is feasible that an excess of neuregulin could outweigh the effect of HER3-targeting.

While this study provides insights into the potential benefits of HER3-targeted HBP nanomedicines for breast cancer BM, further optimization is conceivable. Efficacy could be improved by using a higher concentration of HBP-DOX or substituting the DOX payload with other/combination of cytotoxic or radiotherapeutic payloads. These are feasible approaches since HER3-HBP-DOX did not induce severe toxicity associated with free DOX.

Notably, our study showed that HBP-DOX was well tolerated, and the mice did not experience chemotherapy-associated cardiomyopathy. Anthracyclines, such as doxorubicin, affect fertility by damaging ovarian follicles and depleting oocyte reserves.^59^ As contemporary treatments have significantly extended the survival of cancer patients, preserving fertility has become a concern for patients who wish to conceive.^60^ We showed that HBP-DOX has less chemotherapy-associated effects on the ovarian oocyte reserves, suggesting that it may have a lesser impact on fertility.

Last, the heterogeneously permeable BBB can limit tumor cells exposure to drug and overall treatment efficacy.^61^ Apart from modifying the payload, it is possible to explore approaches that simultaneously enhance the BBB permeability to promote drug access and accumulation. Studies have demonstrated the BBB can be transiently permeabilized to promote drug uptake via hypertonic chemical osmotic modulation,^62^ potassium channel modulation^63^ or microbubble-focused ultrasound.^64^ Others have facilitated BBB crossing by targeting nanomedicine to receptors expressed on brain vascular endothelial cells such as LRP1.^65^

## Conclusions

We demonstrated the theranostic potential of HBP nanomedicine for improving treatment of BM. This proof of concept study shows that DOX delivered via targeted-HBP nanomedicines is better tolerated than free DOX at the same dosage and that HER3-targeting improved the BM outcome. Using PET/MR imaging, we observed tumor-specific localization of nanoparticles with the ability to cross the BBB in this BM model, evident in these images. We further demonstrated significantly reduced systemic off-target effects of the theranostic nanomedicines compared to free chemotherapy, a factor potentially key to their implementation as therapeutic efficacy. In conclusion, delivery of therapeutics via the HBP nanoparticle shows great promise in improving the treatment of brain metastases; however, drug loading and targeting ligand choice are critical to resulting efficacy.

## Supporting Information Available

Amino acid sequences of the HER3 bsAb used in this study, the imaging parameters used for IVIS in vivo bioluminescent and fluorescent imaging, T2-MRI, and autoradiography, the schema and description of the synthesis of the HBP, the nuclear magnetic resonance confirm characterization of: the components in HBP with Cy5, the comparison of the before and after deprotection of TBMC BOC group, and the incorporation of DOX to HBP, the UV–vis spectroscopy confirm characterization of DOX and Cy5 attachment, the dialysis of free DOX in pH 5.5, 6.8, and 7.4, the Western blot confirming HER3 expression in cell lines, the cytotoxicity of HBP formulations to BT474 across a range of concentrations and the intracranial tumor volume after 3 weeks of treatment (PDF)

## Abbreviations

BM: Brain metastasis
NRG1: Neuregulin
DOX: Doxorubicin
HBP: Hyperbranched polymer
HER: Human epidermal growth factor receptor
PET/MR: Positron emission tomography/magnetic resonance

## References

[ref1] Kuksis M.; Gao Y.; Tran W.; Hoey C.; Kiss A.; Komorowski A. S.; Dhaliwal A. J.; Sahgal A.; Das S.; Chan K. K.; et al. The incidence of brain metastases among patients with metastatic breast cancer: a systematic review and meta-analysis. Neuro. Oncol. 2021, 23 (6), 894–904. 10.1093/neuonc/noaa285. 33367836 PMC8168821

[ref2] Cagney D. N.; Martin A. M.; Catalano P. J.; Brown P. D.; Alexander B. M.; Lin N. U.; Aizer A. A. Implications of Screening for Brain Metastases in Patients With Breast Cancer and Non-Small Cell Lung Cancer. JAMA Oncol. 2018, 4 (7), 1001–1003. 10.1001/jamaoncol.2018.0813. 29799956 PMC6145731

[ref3] McTyre E.; Scott J.; Chinnaiyan P. Whole brain radiotherapy for brain metastasis. Surg Neurol Int. 2013, 4, S236–S244. 10.4103/2152-7806.111301. 23717795 PMC3656558

[ref4] Gaspar L.; Scott C.; Rotman M.; Asbell S.; Phillips T.; Wasserman T.; McKenna W. G.; Byhardt R. Recursive partitioning analysis (RPA) of prognostic factors in three Radiation Therapy Oncology Group (RTOG) brain metastases trials. Int. J. Radiat. Oncol. Biol. Phys. 1997, 37 (4), 745–751. 10.1016/S0360-3016(96)00619-0. 9128946

[ref5] Nieder C.; Grosu A. L.; Astner S.; Thamm R.; Molls M. Integration of chemotherapy into current treatment strategies for brain metastases from solid tumors. Radiation Oncology 2006, 1 (1), 19 10.1186/1748-717X-1-19. 16800900 PMC1523351

[ref6] van den Bent M. J. The role of chemotherapy in brain metastases. Eur. J. Cancer 2003, 39 (15), 2114–2120. 10.1016/S0959-8049(03)00577-X. 14522368

[ref7] aLim M.; Puttick S.; Houston Z. H.; Thurecht K. J.; Kalita-de Croft P.; Mahler S.; Rose S. E.; Jeffree R. L.; Mazzieri R.; Dolcetti R.; et al. Innovative Therapeutic Strategies for Effective Treatment of Brain Metastases. Int. J. Mol. Sci. 2019, 20 (6), 1280 10.3390/ijms20061280. 30875730 PMC6471202

[ref8] Kamaly N.; Yameen B.; Wu J.; Farokhzad O. C. Degradable Controlled-Release Polymers and Polymeric Nanoparticles: Mechanisms of Controlling Drug Release. Chem. Rev. 2016, 116 (4), 2602–2663. 10.1021/acs.chemrev.5b00346. 26854975 PMC5509216

[ref9] Hu Y.-B.; Dammer E. B.; Ren R.-J.; Wang G. The endosomal-lysosomal system: from acidification and cargo sorting to neurodegeneration. Transl. Neurodegener. 2015, 4, 18–18. 10.1186/s40035-015-0041-1. 26448863 PMC4596472

[ref10] Matsumura Y.; Maeda H. A new concept for macromolecular therapeutics in cancer chemotherapy: mechanism of tumoritropic accumulation of proteins and the antitumor agent smancs. Cancer Res. 1986, 46, 6387–6392. 2946403

[ref11] Clemons T. D.; Singh R.; Sorolla A.; Chaudhari N.; Hubbard A.; Iyer K. S. Distinction Between Active and Passive Targeting of Nanoparticles Dictate Their Overall Therapeutic Efficacy. Langmuir 2018, 34 (50), 15343–15349. 10.1021/acs.langmuir.8b02946. 30441895

[ref12] Lim M.; Nguyen T. H.; Niland C.; Reid L. E.; Jat P. S.; Saunus J. M.; Lakhani S. R. Landscape of Epidermal Growth Factor Receptor Heterodimers in Brain Metastases. Cancers 2022, 14 (3), 533 10.3390/cancers14030533. 35158800 PMC8833370

[ref13] aBarros F. F.; Abdel-Fatah T. M.; Moseley P.; Nolan C. C.; Durham A. C.; Rakha E. A.; Chan S.; Ellis I. O.; Green A. R. Characterisation of HER heterodimers in breast cancer using in situ proximity ligation assay. Breast Cancer Res. Treat. 2014, 144 (2), 273–285. 10.1007/s10549-014-2871-4. 24557338

[ref14] Da Silva L.; Simpson P. T.; Smart C. E.; Cocciardi S.; Waddell N.; Lane A.; Morrison B. J.; Vargas A. C.; Healey S.; Beesley J.; et al. HER3 and downstream pathways are involved in colonization of brain metastases from breast cancer. Breast cancer research: BCR 2010, 12 (4), R46 10.1186/bcr2603. 20604919 PMC2949633

[ref15] Soltoff S. P.; Carraway K. L. III; Prigent S. A.; Gullick W. G.; Cantley L. C. ErbB3 is involved in activation of phosphatidylinositol 3-kinase by epidermal growth factor. Mol. Cell. Biol. 1994, 14 (6), 3550–3558. 10.1128/MCB.14.6.3550. 7515147 PMC358722

[ref16] aSliwkowski M. X.; Schaefer G.; Akita R. W.; Lofgren J. A.; Fitzpatrick V. D.; Nuijens A.; Fendly B. M.; Cerione R. A.; Vandlen R. L.; Carraway K. L. III Coexpression of erbB2 and erbB3 proteins reconstitutes a high affinity receptor for heregulin. J. Biol. Chem. 1994, 269 (20), 14661–14665. 10.1016/S0021-9258(17)36676-0. 7514177

[ref17] Kodack D. P.; Askoxylakis V.; Ferraro G. B.; Sheng Q.; Badeaux M.; Goel S.; Qi X.; Shankaraiah R.; Cao Z. A.; Ramjiawan R. R. The brain microenvironment mediates resistance in luminal breast cancer to PI3K inhibition through HER3 activation. Sci. Transl. Med. 2017, 9 (391), eaal4682 10.1126/scitranslmed.aal4682. 28539475 PMC5917603

[ref18] Swain S. M.; Baselga J.; Miles D.; Im Y. H.; Quah C.; Lee L. F.; Cortes J. Incidence of central nervous system metastases in patients with HER2-positive metastatic breast cancer treated with pertuzumab, trastuzumab, and docetaxel: results from the randomized phase III study CLEOPATRA. Ann. Oncol. 2014, 25 (6), 1116–1121. 10.1093/annonc/mdu133. 24685829 PMC4037862

[ref19] Saunus J. M.; Quinn M. C.; Patch A. M.; Pearson J. V.; Bailey P. J.; Nones K.; McCart Reed A. E.; Miller D.; Wilson P. J.; Al-Ejeh F.; et al. Integrated genomic and transcriptomic analysis of human brain metastases identifies alterations of potential clinical significance. J. Pathol 2015, 237 (3), 363–378. 10.1002/path.4583. 26172396

[ref20] Semsarilar M.; Ladmiral V.; Blanazs A.; Armes S. P. Anionic Polyelectrolyte-Stabilized Nanoparticles via RAFT Aqueous Dispersion Polymerization. Langmuir 2012, 28 (1), 914–922. 10.1021/la203991y. 22115201

[ref21] Janowicz P. W.; Houston Z. H.; Bunt J.; Fletcher N. L.; Bell C. A.; Cowin G.; Howard C. B.; Taslima D.; Westra van Holthe N.; Prior A.; et al. Understanding nanomedicine treatment in an aggressive spontaneous brain cancer model at the stage of early blood brain barrier disruption. Biomaterials 2022, 283, 121416 10.1016/j.biomaterials.2022.121416. 35217483

[ref22] Fletcher N. L.; Prior A.; Choy O.; Humphries J.; Huda P.; Ghosh S.; Houston Z. H.; Bell C. A.; Thurecht K. J. Pre-targeting of polymeric nanomaterials to balance tumour accumulation and clearance. Chem. Commun. 2022, 58 (57), 7912–7915. 10.1039/D2CC02443H. 35726903

[ref23] Mills J. A.; Humphries J.; Simpson J. D.; Sonderegger S. E.; Thurecht K. J.; Fletcher N. L. Modulating Macrophage Clearance of Nanoparticles: Comparison of Small-Molecule and Biologic Drugs as Pharmacokinetic Modifiers of Soft Nanomaterials. Mol. Pharmaceutics 2022, 19 (11), 4080–4097. 10.1021/acs.molpharmaceut.2c00528. 36069540

[ref24] Mirschberger C.; Schiller C. B.; Schräml M.; Dimoudis N.; Friess T.; Gerdes C. A.; Reiff U.; Lifke V.; Hoelzlwimmer G.; Kolm I.; et al. RG7116, a therapeutic antibody that binds the inactive HER3 receptor and is optimized for immune effector activation. Cancer Res. 2013, 73 (16), 5183–5194. 10.1158/0008-5472.CAN-13-0099. 23780344

[ref25] Lefranc M. P.; Giudicelli V.; Duroux P.; Jabado-Michaloud J.; Folch G.; Aouinti S.; Carillon E.; Duvergey H.; Houles A.; Paysan-Lafosse T.; et al. IMGT®, the international ImMunoGeneTics information system® 25 years on. Nucleic Acids Res. 2015, 43, D413–D422. 10.1093/nar/gku1056. 25378316 PMC4383898

[ref26] Howard C. B.; Fletcher N.; Houston Z. H.; Fuchs A. V.; Boase N. R. B.; Simpson J. D.; Raftery L. J.; Ruder T.; Jones M. L.; de Bakker C. J.; et al. Overcoming Instability of Antibody-Nanomaterial Conjugates: Next Generation Targeted Nanomedicines Using Bispecific Antibodies. Adv. Healthcare Mater. 2016, 5 (16), 2055–2068. 10.1002/adhm.201600263. 27283923

[ref27] Houston Z. H.; Bunt J.; Chen K.-S.; Puttick S.; Howard C. B.; Fletcher N. L.; Fuchs A. V.; Cui J.; Ju Y.; Cowin G.; et al. Understanding the Uptake of Nanomedicines at Different Stages of Brain Cancer Using a Modular Nanocarrier Platform and Precision Bispecific Antibodies. ACS Central Science 2020, 6 (5), 727–738. 10.1021/acscentsci.9b01299. 32490189 PMC7256936

[ref28] Bankhead P.; Loughrey M. B.; Fernández J. A.; Dombrowski Y.; McArt D. G.; Dunne P. D.; McQuaid S.; Gray R. T.; Murray L. J.; Coleman H. G.; et al. QuPath: Open source software for digital pathology image analysis. Sci. Rep. 2017, 7 (1), 16878 10.1038/s41598-017-17204-5. 29203879 PMC5715110

[ref29] Zhao Y.; Fletcher N. L.; Liu T.; Gemmell A. C.; Houston Z. H.; Blakey I.; Thurecht K. J. *In vivo* therapeutic evaluation of polymeric nanomedicines: effect of different targeting peptides on therapeutic efficacy against breast cancer. Nanotheranostics 2018, 2 (4), 360–370. 10.7150/ntno.27142. 30324082 PMC6170333

[ref30] Lim M.; Fletcher N.; McCart Reed A.; Flint M.; Thurecht K.; Saunus J.; Lakhani S. R. Modeling Brain Metastasis by Internal Carotid Artery Injection of Cancer Cells. JoVE 2022, (186), e64216 10.3791/64216. 35993751

[ref31] Kikinis R.; Pieper S. 3D Slicer as a tool for interactive brain tumor segmentation. Annu. Int. Conf. IEEE Eng. Med. Biol. Soc. 2011, 2011, 6982–6984. 10.1109/iembs.2011.6091765. 22255945 PMC3991434

[ref32] Fletcher N. L.; Houston Z. H.; Simpson J. D.; Veedu R. N.; Thurecht K. J. Designed multifunctional polymeric nanomedicines: long-term biodistribution and tumour accumulation of aptamer-targeted nanomaterials. Chem. Commun. 2018, 54 (82), 11538–11541. 10.1039/C8CC05831H. 30182121

[ref33] Chen L.; Simpson J. D.; Fuchs A. V.; Rolfe B. E.; Thurecht K. J. Effects of Surface Charge of Hyperbranched Polymers on Cytotoxicity, Dynamic Cellular Uptake and Localization, Hemotoxicity, and Pharmacokinetics in Mice. Mol. Pharmaceutics 2017, 14 (12), 4485–4497. 10.1021/acs.molpharmaceut.7b00611. 29116801

[ref34] Pearce A. K.; Rolfe B. E.; Russell P. J.; Tse B. W. C.; Whittaker A. K.; Fuchs A. V.; Thurecht K. J. Development of a polymer theranostic for prostate cancer. Polym. Chem. 2014, 5 (24), 6932–6942. 10.1039/C4PY00999A.

[ref35] Zhao Y.; Fletcher N. L.; Gemmell A.; Houston Z. H.; Howard C. B.; Blakey I.; Liu T.; Thurecht K. J. Investigation of the Therapeutic Potential of a Synergistic Delivery System through Dual Controlled Release of Camptothecin-Doxorubicin. Advanced Therapeutics 2020, 3 (6), 1900202 10.1002/adtp.201900202.

[ref36] Rizvi S. A. A.; Saleh A. M. Applications of nanoparticle systems in drug delivery technology. Saudi Pharm. J. 2018, 26 (1), 64–70. 10.1016/j.jsps.2017.10.012. 29379334 PMC5783816

[ref37] Cui J.; Ju Y.; Houston Z. H.; Glass J. J.; Fletcher N. L.; Alcantara S.; Dai Q.; Howard C. B.; Mahler S. M.; Wheatley A. K.; et al. Modulating Targeting of Poly(ethylene glycol) Particles to Tumor Cells Using Bispecific Antibodies. Adv. Healthcare Mater. 2019, 8 (9), 1801607 10.1002/adhm.201801607. 30868751

[ref38] Ling W. L.; Lua W. H.; Poh J. J.; Yeo J. Y.; Lane D. P.; Gan S. K. Effect of VH-VL Families in Pertuzumab and Trastuzumab Recombinant Production, Her2 and FcγIIA Binding. Front. Immunol. 2018, 9, 469 10.3389/fimmu.2018.00469. 29593727 PMC5857972

[ref39] Schoeberl B.; Pace E. A.; Fitzgerald J. B.; Harms B. D.; Xu L.; Nie L.; Linggi B.; Kalra A.; Paragas V.; Bukhalid R.; et al. Therapeutically targeting ErbB3: a key node in ligand-induced activation of the ErbB receptor-PI3K axis. Sci. Signal 2009, 2 (77), ra31 10.1126/scisignal.2000352. 19567914

[ref40] aSivaram A. J.; Wardiana A.; Alcantara S.; Sonderegger S. E.; Fletcher N. L.; Houston Z. H.; Howard C. B.; Mahler S. M.; Alexander C.; Kent S. J.; et al. Controlling the Biological Fate of Micellar Nanoparticles: Balancing Stealth and Targeting. ACS Nano 2020, 14 (10), 13739–13753. 10.1021/acsnano.0c06033. 32936613

[ref41] Talbot S. R.; Biernot S.; Bleich A.; van Dijk R. M.; Ernst L.; Häger C.; Helgers S. O. A.; Koegel B.; Koska I.; Kuhla A.; et al. Defining body-weight reduction as a humane endpoint: a critical appraisal. Lab Anim. 2020, 54 (1), 99–110. 10.1177/0023677219883319. 31665969

[ref42] aMinotti G.; Menna P.; Salvatorelli E.; Cairo G.; Gianni L. Anthracyclines: molecular advances and pharmacologic developments in antitumor activity and cardiotoxicity. Pharmacol Rev. 2004, 56 (2), 185–229. 10.1124/pr.56.2.6. 15169927

[ref43] Florescu M.; Cinteza M.; Vinereanu D. Chemotherapy-induced Cardiotoxicity. Maedica 2013, 8 (1), 59–67. 24023601 PMC3749765

[ref44] aSchimmel K. J. M.; Richel D. J.; van den Brink R. B. A.; Guchelaar H.-J. Cardiotoxicity of cytotoxic drugs. Cancer Treat Rev. 2004, 30 (2), 181–191. 10.1016/j.ctrv.2003.07.003. 15023436

[ref45] Song K.; Ma W.; Huang C.; Ding J.; Cui D.; Zhang M. Expression Pattern of Mouse Vasa Homologue (MVH) in the Ovaries of C57BL/6 Female Mice. Med. Sci. Monit. 2016, 22, 2656–2663. 10.12659/MSM.899830. 27460133 PMC4973802

[ref46] Venur V. A.; Leone J. P. Targeted Therapies for Brain Metastases from Breast Cancer. Int. J. Mol. Sci. 2016, 17 (9), 1543 10.3390/ijms17091543. 27649142 PMC5037817

[ref47] aMurthy R.; Borges V. F.; Conlin A.; Chaves J.; Chamberlain M.; Gray T.; Vo A.; Hamilton E. Tucatinib with capecitabine and trastuzumab in advanced HER2-positive metastatic breast cancer with and without brain metastases: a non-randomised, open-label, phase 1b study. Lancet Oncology 2018, 19 (7), 880–888. 10.1016/S1470-2045(18)30256-0. 29804905

[ref48] Mueller V.; Wardley A.; Paplomata E.; Hamilton E.; Zelnak A.; Fehrenbacher L.; Jakobsen E.; Curtit E.; Boyle F.; Harder Brix E.; et al. Preservation of quality of life in patients with human epidermal growth factor receptor 2-positive metastatic breast cancer treated with tucatinib or placebo when added to trastuzumab and capecitabine (HER2CLIMB trial). Eur. J. Cancer 2021, 153, 223–233. 10.1016/j.ejca.2021.05.025. 34214937

[ref49] Aston W. J.; Hope D. E.; Nowak A. K.; Robinson B. W.; Lake R. A.; Lesterhuis W. J. A systematic investigation of the maximum tolerated dose of cytotoxic chemotherapy with and without supportive care in mice. BMC Cancer 2017, 17 (1), 684–684. 10.1186/s12885-017-3677-7. 29037232 PMC5644108

[ref50] Wallace K. B.; Sardão V. A.; Oliveira P. J. Mitochondrial Determinants of Doxorubicin-Induced Cardiomyopathy. Circ. Res. 2020, 126 (7), 926–941. 10.1161/CIRCRESAHA.119.314681. 32213135 PMC7121924

[ref51] Adams G. P.; Schier R.; McCall A. M.; Simmons H. H.; Horak E. M.; Alpaugh R. K.; Marks J. D.; Weiner L. M. High affinity restricts the localization and tumor penetration of single-chain fv antibody molecules. Cancer Res. 2001, 61 (12), 4750–4755. 11406547

[ref52] Bordeau B. M.; Abuqayyas L.; Nguyen T. D.; Chen P.; Balthasar J. P. Development and Evaluation of Competitive Inhibitors of Trastuzumab-HER2 Binding to Bypass the Binding-Site Barrier. Front. Pharmacol. 2022, 13, 837744 10.3389/fphar.2022.837744. 35250584 PMC8895951

[ref53] aJuweid M.; Neumann R.; Paik C.; Perez-Bacete M. J.; Sato J.; van Osdol W.; Weinstein J. N. Micropharmacology of monoclonal antibodies in solid tumors: direct experimental evidence for a binding site barrier. Cancer Res. 1992, 52 (19), 5144–5153. 1327501

[ref54] aUghachukwu P.; Unekwe P. Efflux pump-mediated resistance in chemotherapy. Ann. Med. Health Sci. Res. 2012, 2 (2), 191–198. 10.4103/2141-9248.105671. 23439914 PMC3573517

[ref55] Stefanski C. D.; Keffler K.; McClintock S.; Milac L.; Prosperi J. R. APC loss affects DNA damage repair causing doxorubicin resistance in breast cancer cells. Neoplasia 2019, 21 (12), 1143–1150. 10.1016/j.neo.2019.09.002. 31759252 PMC6872841

[ref56] Li L.-Y.; Guan Y.-d.; Chen X.-S.; Yang J.-M.; Cheng Y. DNA Repair Pathways in Cancer Therapy and Resistance. Front. Pharmacol. 2021, 11, 629266–629266. 10.3389/fphar.2020.629266. 33628188 PMC7898236

[ref57] Lopergolo A.; Tavecchio M.; Lisanti S.; Ghosh J. C.; Dohi T.; Faversani A.; Vaira V.; Bosari S.; Tanigawa N.; Delia D.; et al. Chk2 phosphorylation of survivin-DeltaEx3 contributes to a DNA damage-sensing checkpoint in cancer. Cancer Res. 2012, 72 (13), 3251–3259. 10.1158/0008-5472.CAN-11-4035. 22586065 PMC3695608

[ref59] Xiao S.; Zhang J.; Liu M.; Iwahata H.; Rogers H. B.; Woodruff T. K. Doxorubicin Has Dose-Dependent Toxicity on Mouse Ovarian Follicle Development, Hormone Secretion, and Oocyte Maturation. Toxicol. Sci. 2017, 157 (2), 320–329. 10.1093/toxsci/kfx047. 28329872 PMC6074798

[ref60] aJohnson F. E.; Liebscher G. J.; LaRegina M. C.; Tolman K. C. Preservation of fertility following doxorubicin administration in the rat. Surg. Oncol. 1992, 1 (2), 145–150. 10.1016/0960-7404(92)90027-I. 1341245

[ref61] Lockman P. R.; Mittapalli R. K.; Taskar K. S.; Rudraraju V.; Gril B.; Bohn K. A.; Adkins C. E.; Roberts A.; Thorsheim H. R.; Gaasch J. A.; et al. Heterogeneous blood-tumor barrier permeability determines drug efficacy in experimental brain metastases of breast cancer. Clin. Cancer Res. 2010, 16 (23), 5664–5678. 10.1158/1078-0432.CCR-10-1564. 20829328 PMC2999649

[ref62] Rapoport S. I. Osmotic opening of the blood-brain barrier: principles, mechanism, and therapeutic applications. Cell Mol. Neurobiol 2000, 20 (2), 217–230. 10.1023/A:1007049806660. 10696511 PMC11537517

[ref63] Miao T.; Ju X.; Zhu Q.; Wang Y.; Guo Q.; Sun T.; Lu C.; Han L. Nanoparticles Surmounting Blood-Brain Tumor Barrier Through Both Transcellular and Paracellular Pathways to Target Brain Metastases. Adv. Funct. Mater. 2019, 29 (27), 1900259 10.1002/adfm.201900259.

[ref64] Chen K. T.; Wei K. C.; Liu H. L. Theranostic Strategy of Focused Ultrasound Induced Blood-Brain Barrier Opening for CNS Disease Treatment. Front. Pharmacol. 2019, 10, 86 10.3389/fphar.2019.00086. 30792657 PMC6374338

[ref65] Guo Q.; Zhu Q.; Miao T.; Tao J.; Ju X.; Sun Z.; Li H.; Xu G.; Chen H.; Han L. LRP1-upregulated nanoparticles for efficiently conquering the blood-brain barrier and targetedly suppressing multifocal and infiltrative brain metastases. J. Controlled Release 2019, 303, 117–129. 10.1016/j.jconrel.2019.04.031. 31026546

