# Supplementary material for: Targeted Hyperbranched Nanoparticles for Delivery of Doxorubicin in Breast Cancer Brain Metastasis
Source: Mol Pharm. 2023 Nov 16;20(12):6169–83. doi: 10.1021/acs.molpharmaceut.3c00558 (PMC10699306; doi:10.1021/acs.molpharmaceut.3c00558)
Supplement: Supplementary file 1 — mp3c00558_si_001.pdf [file mp3c00558_si_001.pdf]

Supporting information

Targeted Hyperbranched Nanoparticles for Delivery of Doxorubicin in Breast Cancer Brain Metastasis

Malcolm Lim [a§], Nicholas L Fletcher [b,c,d,e§], Jodi M Saunus [a#], Amy E McCart Reed [a], Haarika Chittoory [a], Peter T Simpson [a], Kristofer J Thurecht [b,c,d,e], Sunil R Lakhani [a,f*].

[a] UQ Centre for Clinical Research, Faculty of Medicine, The University of Queensland, Brisbane, Herston, Queensland, 4006, Australia

[b] Centre for Advanced Imaging, The University of Queensland, Brisbane, St Lucia, Queensland, 4072, Australia.

[c] Australian Research Council Training Centre for Innovation in Biomedical Imaging Technology, The University of Queensland, Brisbane, St Lucia, Queensland, 4072, Australia.

[d] Australian Research Council Centre of Excellence in Convergent Bio-Nano Science and Technology, The University of Queensland, Brisbane, St Lucia, Queensland, 4072, Australia

[e] Australian Institute for Bioengineering and Nanotechnology, The University of Queensland, Brisbane, St Lucia, Queensland, 4072, Australia

[f] Pathology Queensland, Royal Brisbane and Women's Hospital, Herston, Queensland, 4006, Australia

*Corresponding author email: s.lakhani@uq.edu.au

Table S1 Amino acid sequences of the HER3 bsAb used in this study.

Amino acid sequences for synthesis of bsAb incorporated mammalian leader sequence (black), histidine-tag (yellow), lumretuzumab-scFv sequence (grey), (G4S)3 linker sequence (green), anti-PEG scFv (blue) and Myc tag (purple).

Lumretuzumab-PEG-BsAb

**MGWSCIILFLVATATGVHSHHHHHHQVQLVQSGAEVKKPGASVKVSCKASGYTFRS
SYISWVRQAPGQGLEWMGWIYAGTGSPSYNQKLQGRVTMTTDTSTSTAYMELRSLR
SDDTAVYYCARHRDYYSNSLTYWGQGTLVTVSSGGGGSGGGGSGGGGSDIVMTQS
PDSLAVSLGERATINCKSSQSVLNSGNQKNYLTWYQQKPGQPPKLLIYWASTRESG
VPDRFSGSGSGTDFTLTISSLQAEDVAVYYCQSDYSYPYTFGQGTKLEIKSGGGGSE
VKLEESGGGLVQPGGSMKLSCVASGFTFSNYWMNWVRQSPEKGLEWVTEIRSKSN
NYATHYAESVKGRFTISRDDSKGSVYLQMNNLRAEDTGIYYCSNRYYWGQGTLVTVS
AGGGGSGGGGSGGGGSDIVMTQSHKFMSTSVRDRVTITCKASQDVNTSVAWYQQK
PGQSPKLVIYWASTRHTGVPDRFTGSGSGTDFTLTISNVQSEDLADYFCLQYINYPYT
FGGGTKLEIKEQKLISEEDLN**

Table S2 Imaging settings

**IVIS scan settings**	**Type of imaging**		
**Parameters**	**Optical (in vivo)**	**DOX (ex vivo)**	**Cy5 (ex vivo)**
Exposure value	*Auto*	*Auto*	*Auto*
F-stop	*1*	*2*	*2*
Binning	*Medium*	*Medium*	*Medium*
Field of view	*C*	*A*	*A*
Excitation	*Blocked*	*480*	*600*
Emission	*Open*	*620*	*670*

**T2 MRI scan settings**	**Plane**	
**Parameters**	**Coronal**	**Transverse**
Slices	*23x0.6mm*	*22x0.6mm*
Field of View	*19x19mm*	*19x19mm*
In-plane resolution	*98x98µm*	*98x98µm*
Repetition time	*2200ms*	*2650ms*
Echo time	*35ms*	*35ms*
Averages	*2*	*2*
Acquisition time	*5m19s*	*5m19s*

**T1 MRI scan settings**	**Plane**	
**Parameters**	**Coronal**	**Transverse**
Slices	*23x0.6mm*	*22x0.6mm*
Field of View	*18x30mm*	*18x30mm*
In-plane resolution	*98x98µm*	*98x98µm*
Repetition time	*12*	*12*
Echo time	*2*	*2*
Averages	*3*	*3*
Acquisition time	*28m30s*	*28m30s*
Flip angle	*21*	*21*

**Autoradiography settings**		
**Parameters**	**Phosphor**	**Cy5 (635nm)**
**Pixel size**	50µm	50
**Sensitivity**	1000	Auto

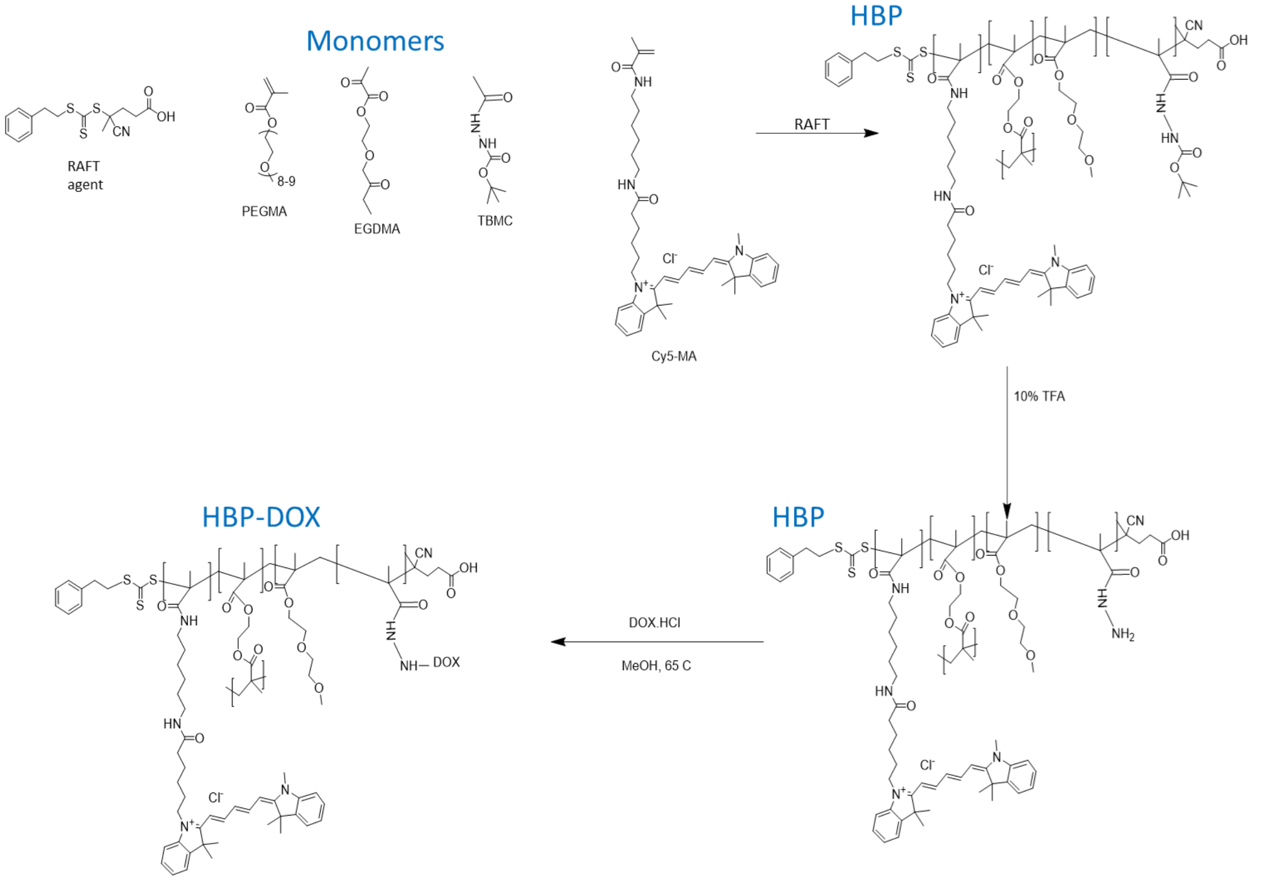

Figure S1 Schema for hyperbranched polymer (HBP-DOX) synthesis.

Poly(ethylene glycol) methacrylate (PEGMA) was utilized as the primary monomer component to impart solubility and biological compatibility. Et hylene glycol dimethacrylate (EGDMA) and Cyanine 5 methacrylamide (Cy5MA) were included at 6 and 0.6 mol% feed ratio relative to PEGMA as branching agents and fluorescent tags respectively. The BOC-protected hydrazide methacrylate was also incorporated at 25 mol% relative to PEGMA for subsequent DOX loading steps and polymerization utilized 4,4′-azobis (cyanovaleric acid) as an initiator and 4-Cyano-4-(phenylcarbonothioylthio)pentanoic acid (TBMC) as the RAFT agent. Following RAFT polymerization, the HBP was then modified to incorporate azido-chain end functionality and the BOC protecting groups were removed through a trifluoracetic acid (10% TFA) deprotection step. DOX (DOX.HCl) was then incorporated under reflux conditions in methanol (MeOH) to incorporate DOX through a cleavable hydrazone linkage forming HBP-DOX.

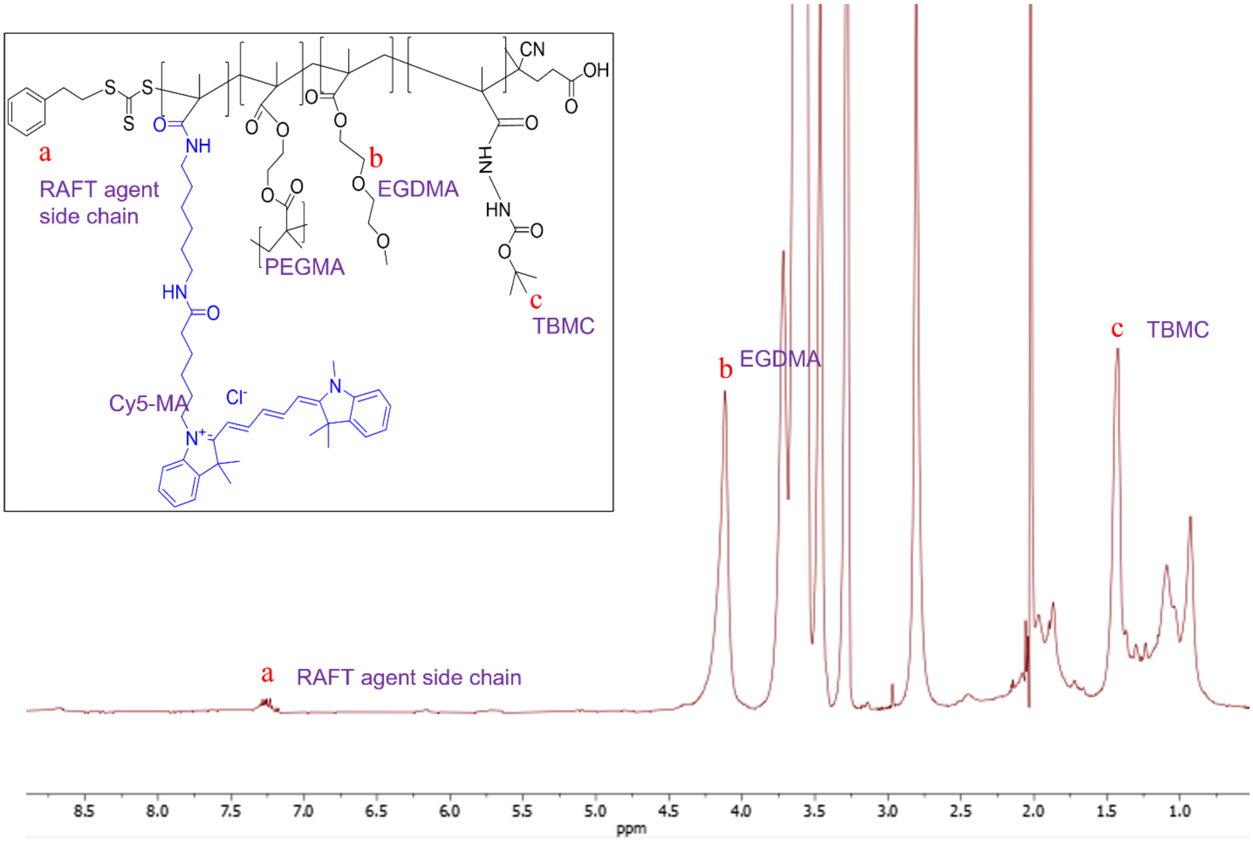

Figure S2 Nuclear magnetic resonance (NMR) analysis of HBP with Cy5.

NMR shows the peaks of the HBP components (RAFT agent side chain, EGDMA,TBMC).

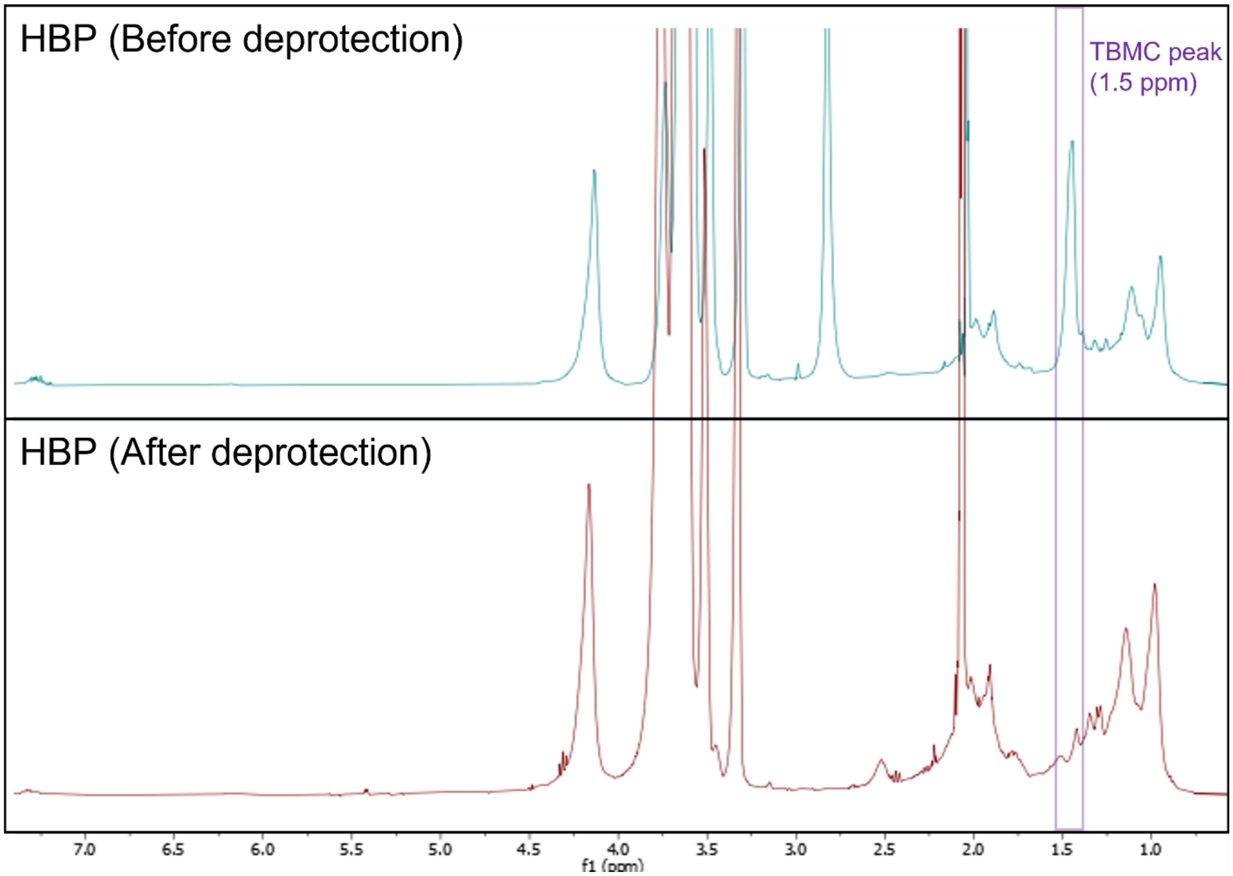

Figure S3 HBP with Cy5 after deprotection and removal of TBMC BOC group.

Figure shows nuclear magnetic resonance analysis of HBP before (top chart) and after (below chart) deprotection.

Comparing the charts confirmed that the TBMC peak at ~1.5 ppm was removed.

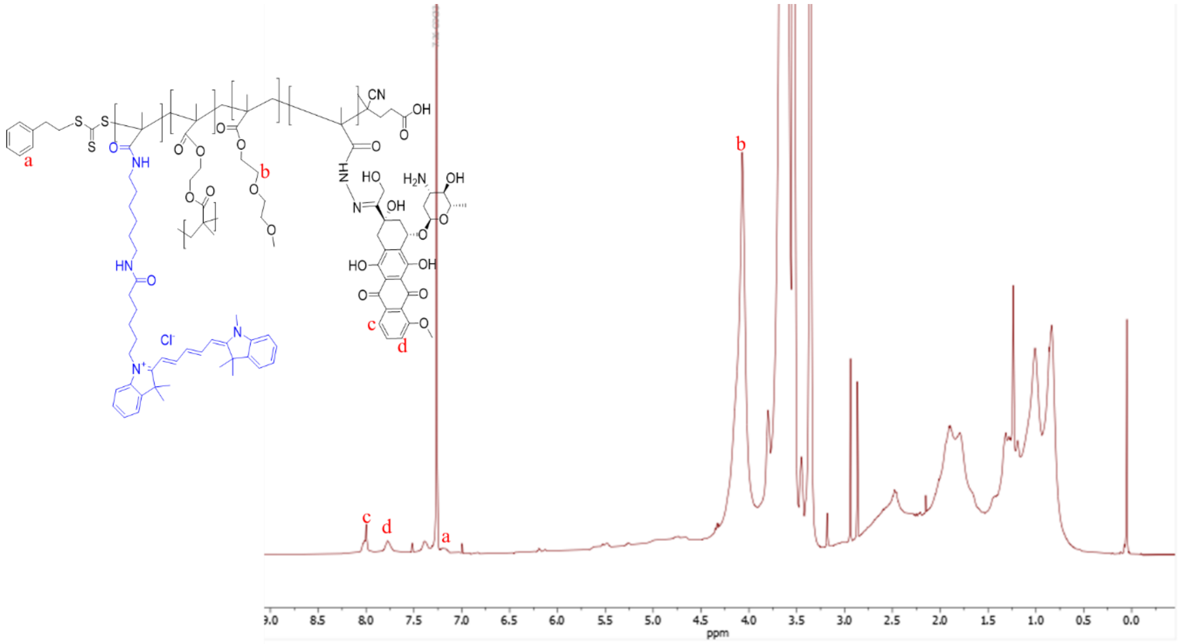

Figure S4 Final HBP with DOX functionalisation.

DOX peaks at *c* and *d* indicating the formation of hydrazone bond linking HBP and DOX.

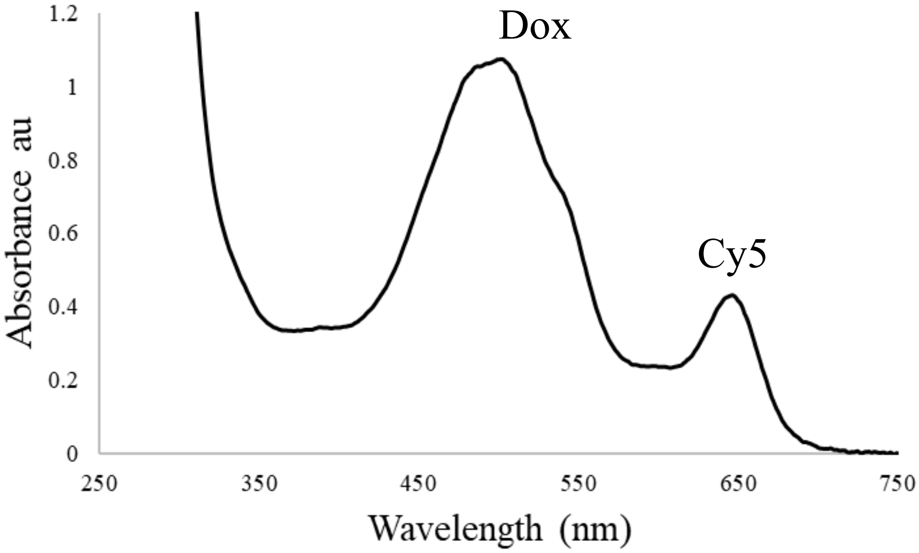

Figure S5 UV-Visible spectroscopy of HBP-DOX with Cy5.

UV-Vis analysis confirmed the attachment of DOX and Cy5 that peaked at 480 nm and 650nm respectively.

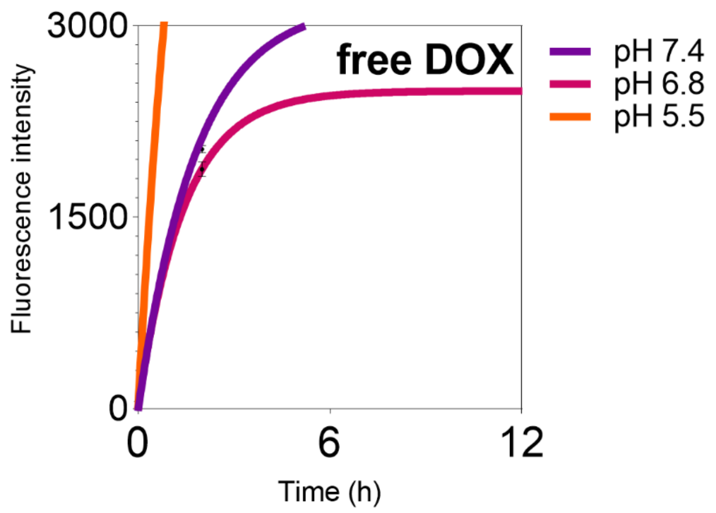

Figure S6 Dialysis of free DOX.

The graph shows the increasing fluorescence intensity of doxorubicin as the unbound drug diffuses into external buffer over time at 37°C. The pH values of 5.5, 6.8 and 7.4, represent the acidity levels of endosomal compartments, tumor tissue microenvironment and normal tissue respectively. Mean +/- standard error shown. The Y-axis represents fluorescence intensity of doxorubicin at emission wavelength of 590nm.

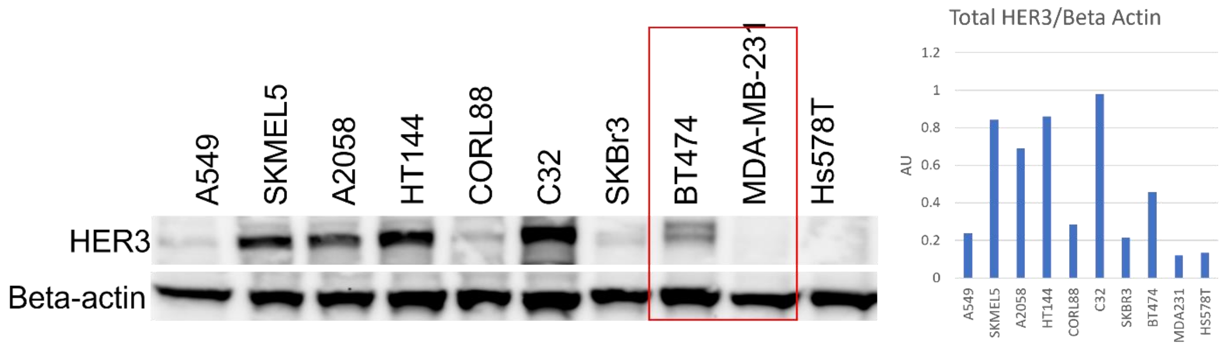

Figure S7 Western analysis of a panel of cancer cell lines for HER3 protein expression.

Western blots for HER3 using whole-cell extracts from a panel of cancer cell lines. Amongst these, the breast cancer cell line, BT474, expressed moderate amount of HER3. Data showed that BT474 expressed higher amounts of HER3 protein compared to MDA-MB-231 (red boxed). This is illustrated by densitometry plot on the right.

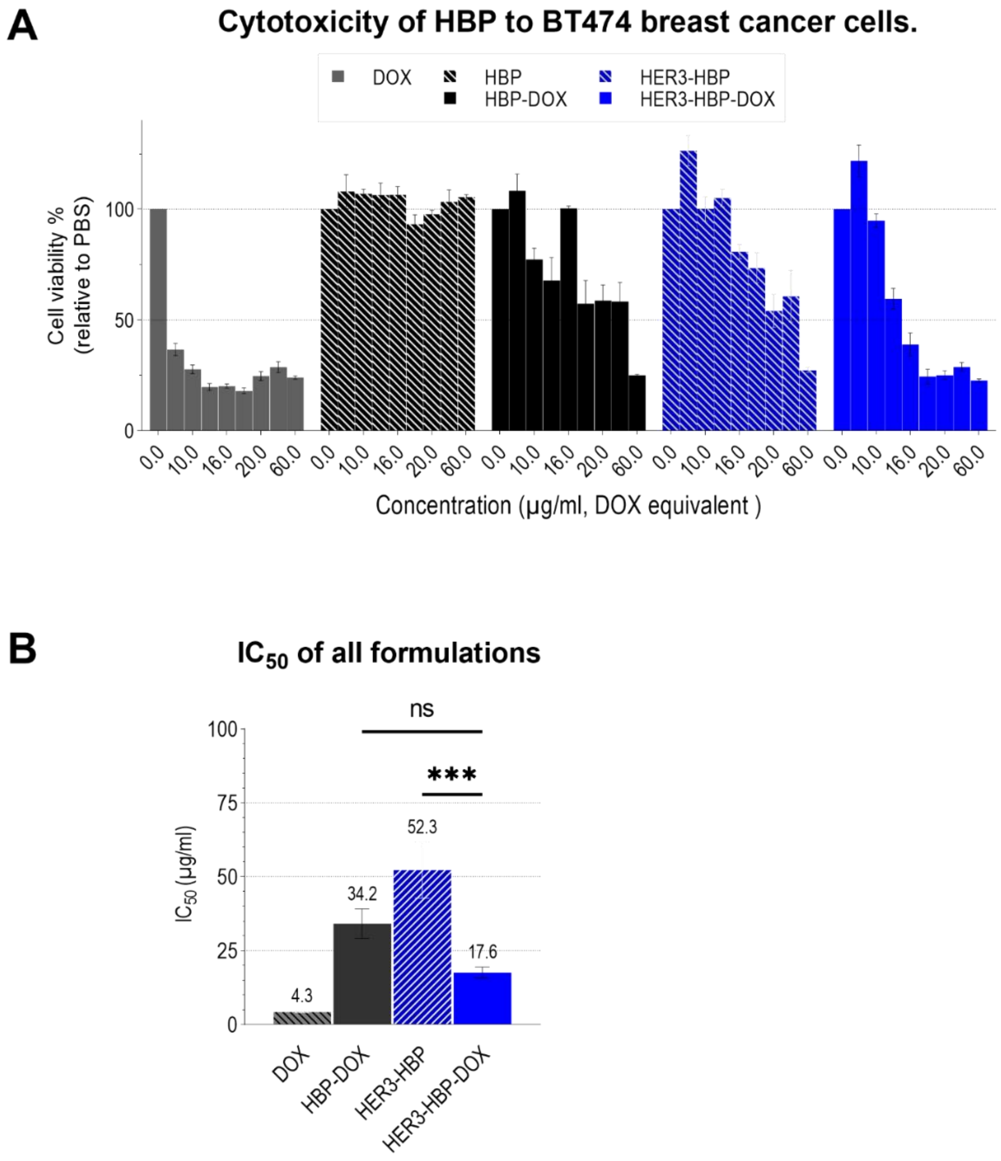

Figure S8 Cytotoxicity of HBP formulations against BT474 cells.

Cell viability for each treatment dosed at a range of concentrations relative to PBS. Significance (***$p<0.001$) derived from ANOVA test. (B) IC50 of various HBP formulations against BT474 cells. Data shown are the average ± standard error of the mean of three independent experiments.

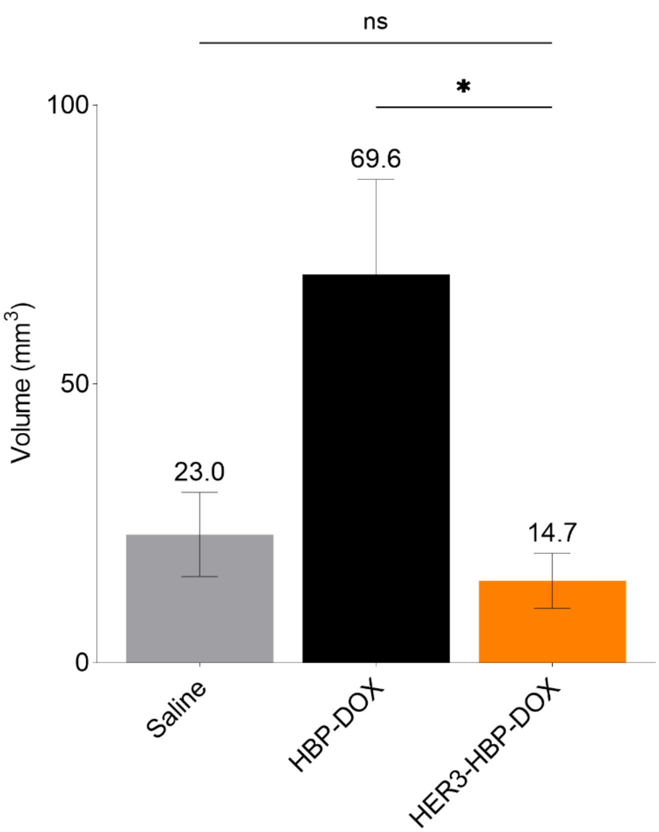

Figure S9 Intracranial tumor volume following 3 weeks of treatment.

T2-weighted MR images of animals were taken following 3 weeks of treatment and the tumor volume were determined by MRI volumetry. At this early timepoint, HER3-HBP-DOX group had smaller brain tumors than saline and HBP-DOX groups (ANOVA test, 0.703 and 0.018 respectively).
